# The subcellular arrangement of alpha-synuclein proteoforms in the Parkinson’s disease brain as revealed by multicolor STED microscopy

**DOI:** 10.1007/s00401-021-02329-9

**Published:** 2021-06-11

**Authors:** Tim E. Moors, Christina A. Maat, Daniel Niedieker, Daniel Mona, Dennis Petersen, Evelien Timmermans-Huisman, Jeroen Kole, Samir F. El-Mashtoly, Liz Spycher, Wagner Zago, Robin Barbour, Olaf Mundigl, Klaus Kaluza, Sylwia Huber, Melanie N. Hug, Thomas Kremer, Mirko Ritter, Sebastian Dziadek, Jeroen J. G. Geurts, Klaus Gerwert, Markus Britschgi, Wilma D. J. van de Berg

**Affiliations:** 1 Department of Anatomy and Neurosciences, Clinical Neuroanatomy and Biobanking, Amsterdam Neuroscience, Amsterdam UMC, Location VU University Medical Center, O2 building, room 13 E11, De Boelelaan 1108, 1081 HZ Amsterdam, The Netherlands; 2 Department of Biophysics, Ruhr-University Bochum, Universitätsstrasse 150, 44801 Bochum, Germany; 3 Roche Pharma Research and Early Development; Neuroscience and Rare Diseases Discovery and Translational Area, Roche Innovation Center Basel, Grenzacherstrasse 124, CH—4070 Basel, Switzerland; 4 Department of Physiology, Amsterdam UMC, Location VU University Medical Center, De Boelelaan 1108, 1081 HZ Amsterdam, The Netherlands; 5 Prothena Biosciences Inc, South San Francisco, CA 94080 USA; 6 Roche Pharma Research and Early Development, Therapeutic Modalities; Large Molecule Research, Roche Innovation Center Munich, Nonnenwald 2, 82377 Penzberg, Germany; 7 Roche Pharma Research and Early Development; Therapeutic Modalities; Small Molecule Research, Roche Innovation Center Basel, Grenzacherstrasse 124, CH—4070 Basel, Switzerland; 8 Roche Diagnostics GmbH, Nonnenwald 2, 82377 Penzberg, Germany; 9 Roche Pharma Research and Early Development; Oncology Discovery and Translational Area, Roche Innovation Center Basel, Grenzacherstrasse 124, Basel, Switzerland; 10 Ann Romney Center for Neurologic Diseases, Department of Neurology, Brigham and Women’s Hospital, Harvard Medical School, Boston, MA 02115 USA

**Keywords:** Alpha-synuclein, Parkinson's disease, Lewy bodies, Post-translational modifications, Super-resolution microscopy, Post-mortem human brain

## Abstract

Various post-translationally modified (PTM) proteoforms of alpha-synuclein (aSyn)—including C-terminally truncated (CTT) and Serine 129 phosphorylated (Ser129-p) aSyn—accumulate in Lewy bodies (LBs) in different regions of the Parkinson’s disease (PD) brain. Insight into the distribution of these proteoforms within LBs and subcellular compartments may aid in understanding the orchestration of Lewy pathology in PD. We applied epitope-specific antibodies against CTT and Ser129-p aSyn proteoforms and different aSyn domains in immunohistochemical multiple labelings on post-mortem brain tissue from PD patients and non-neurological, aged controls, which were scanned using high-resolution 3D multicolor confocal and stimulated emission depletion (STED) microscopy. Our multiple labeling setup highlighted a consistent onion skin-type 3D architecture in mature nigral LBs in which an intricate and structured-appearing framework of Ser129-p aSyn and cytoskeletal elements encapsulates a core enriched in CTT aSyn species. By label-free CARS microscopy we found that enrichments of proteins and lipids were mainly localized to the central portion of nigral aSyn-immunopositive (aSyn+) inclusions. Outside LBs, we observed that 122CTT aSyn+ punctae localized at mitochondrial membranes in the cytoplasm of neurons in PD and control brains, suggesting a physiological role for 122CTT aSyn outside of LBs. In contrast, very limited to no Ser129-p aSyn immunoreactivity was observed in brains of non-neurological controls, while the alignment of Ser129-p aSyn in a neuronal cytoplasmic network was characteristic for brains with (incidental) LB disease. Interestingly, Ser129-p aSyn+ network profiles were not only observed in neurons containing LBs but also in neurons without LBs particularly in donors at early disease stage, pointing towards a possible subcellular pathological phenotype preceding LB formation. Together, our high-resolution and 3D multicolor microscopy observations in the post-mortem human brain provide insights into potential mechanisms underlying a regulated LB morphogenesis.

**Supplementary Information:**

The online version contains supplementary material available at 10.1007/s00401-021-02329-9.

## Introduction

The presence of neuronal inclusions—termed Lewy Bodies (LBs) and Lewy Neurites (LNs)—in predilected brain regions pathologically defines Parkinson’s disease (PD) and dementia with Lewy bodies (DLB). LBs are described as eosinophilic inclusion bodies with different morphologies, typically dependent on brain region (brainstem, limbic or cortical) [40, 70]. The mechanisms determining their formation and morphology remain elusive. LBs and LNs are immunopositive for alpha-synuclein (aSyn), which is one of their major protein components [68], and ultrastructurally characterized by accumulated membranous and organellar material [67]. aSyn is a 14 kDa protein ubiquitously and highly expressed in neurons under physiological conditions. Its enrichment in presynaptic terminals, where aSyn is associated with synaptic vesicles, has been established [7, 25, 31, 49], while more recent studies have reported additional intraneuronal localizations for aSyn, including mitochondria, endoplasmatic reticulum (ER) and Golgi apparatus [7]. The primary sequence of aSyn contains 140 amino acids and is composed of three distinct domains. An important role has been proposed for the lipophilic N-terminus (NT) and non-amyloid-β component domain (NAC domain) in the interaction of aSyn with lipid membranes [7, 18], while the residues 96–140 encompass the negatively charged, acidic C-terminus (CT) of aSyn for which important regulatory roles have been proposed in the interaction of aSyn with other proteins or metal ions [17]. The CT further harbors many sites where aSyn can be post-translationally modified (PTM) [57].

The list of aSyn PTMs detected in the human brain has grown extensively in recent years, which highlights the physicochemical and structural flexibility of aSyn [42, 51]. Some of these PTMs have been implicated in PD pathology—in particular phosphorylation at Serine 129 (Ser129-p) and truncations of the C-terminus (CTT). Ser129-p aSyn and different CTT fragments of aSyn were identified in aqueous buffer insoluble fractions of the DLB brain using mass spectrometry and immuno-based biochemical assays [3, 21, 35]. Among the CTT variants of aSyn most consistently identified in human brain tissue are the truncations at Asp-119 and Asn-122 [3, 35, 44]. Although Ser129-p and CTT aSyn can be detected in low concentrations under physiological circumstances [52], levels of these PTMs are markedly enriched in insoluble tissue fractions of donors with PD or DLB [3, 5, 21, 27, 44]. Although the aggregation state of PTM aSyn in the brain remains unclear, a role of CTT in aSyn aggregation mechanisms has been proposed based on experimental in vitro studies [28, 44, 45, 53, 71]. Moreover, analyses in post-mortem brain tissue of DLB patients and aSyn transgenic mouse brains pointed to a potential role of 122CTT in axonal and synaptic degeneration [13, 23, 24], while these effects were ameliorated by blocking of calpain-mediated cleavage of CT aSyn by overexpressing calpastatin in aSyn transgenic mice [13].

Resultant from these findings a great interest emerged for CTT and Ser129-p aSyn variants as potential biomarkers for PD [11, 66], leading to the development of research tools such as antibodies selectively directed against these aSyn proteoforms. Antibodies against CTT and Ser129-p aSyn readily detect LBs and LNs in PD/DLB brain tissue, supporting their enrichment in pathological aSyn inclusions [3, 27, 62]. However, despite the availability of such tools, more detailed descriptions of the subcellular localization of these PTM aSyn forms in the human brain under physiological and pathological conditions remain sparse. One study showed different localization for antibodies directed against CTT aSyn (syn105) and Ser129-p aSyn (11A5) in midbrain LBs and dystrophic LNs in post-mortem brain-tissue sections of donors with incidental Lewy body disease (iLBD) and PD patients by confocal microscopy. The authors suggested a laminar organization of nigral LBs in which tyrosine hydroxylase and ubiquitin in the core of these inclusions were surrounded by CTT aSyn and Ser129-p, respectively [62]. These observations point to a potential directed organization of the assembly of PTM aSyn and other components within LBs in the post-mortem brain, but require validation and quantification using the same and additional (PTM) aSyn-specific antibodies.

In addition, the subcellular distribution patterns of CTT and Ser129-p aSyn outside inclusions in neurons with or without LBs, and their relevance in normally aged and PD brains remain unclear. Where the resolution of conventional optical imaging methods such as CSLM is limited by diffraction, recently developed super-resolution microscopy techniques such as stimulated depletion microscopy (STED) allow for the sensitive detection and visualization of e.g. antibody-labeled proteins at a subcellular resolution. The combination of epitope-specific antibodies against aSyn proteoforms and different aSyn domains and STED microscopy in relevant human post-mortem brain tissue can contribute to the discovery of previously indiscernible phenotypes of aSyn cytopathology. Such detailed information derived from the human brain can yield important insights into cellular processes involved in LB morphogenesis, provide an important reference that can contribute to the translation of cellular changes in experimental model systems to human brain pathology, and may help guide future aSyn targeting therapies.

In this study, we mapped subcellular immunoreactivity patterns for antibodies selectively directed against Ser129-p and two CTT aSyn variants (119CTT and 122CTT) in neurons in post-mortem brains from PD patients at different disease stages, donors with iLBD, and aged clinically non-neurological control subjects using high-resolution 3D multicolor imaging techniques such as CLSM and multicolor STED microscopy. Antibodies included in this study were either previously published or newly generated, for which information on their generation and initial characterization is provided here. Using these tools, we observed a heterogeneous landscape of neuronal aSyn-immunopositive (aSyn+) features, in which a subset of mature nigral LBs revealed a consistent, structured-appearing onion skin-like architecture replicating and extending previous findings by Prasad et al. [62]. Ser129-p aSyn at the periphery of such LBs is embedded in an intricate framework of cytoskeletal elements that surrounds a core enriched in CTT fragments, proteins, and lipids as shown by label-free coherent anti-Stokes Raman scattering (CARS) microscopy. These findings suggest that the morphology of mature LBs reflects a regulated encapsulation of accumulated proteins and lipids by cytoskeletal material and Ser129-p aSyn. We further demonstrate that punctate cytoplasmic 122CTT immunolabeling in both patients and control subjects localize at mitochondrial membranes, suggesting a physiological role of this variant outside LBs. The alignment of Ser129-p aSyn in a cytoplasmic network was observed in neurons in PD and iLBD—but not control—brains, in neurons containing LBs but also in neurons without apparent inclusion. We observed this subcellular phenotype particularly in iLBD (Braak 3,4), pointing to a possible change in subcellular Ser129-p aSyn distribution preceding LB formation. Together, our observations by high-resolution multicolor microscopy in post-mortem human brain tissue provide novel insights into potential mechanisms underlying a regulated LB morphogenesis in PD.

## Materials and methods

### Post-mortem human brain tissue

Post-mortem human brain tissue from clinically diagnosed and neuropathologically verified donors with advanced PD or DLB as well as non-neurological controls was collected by the Netherlands Brain Bank (www.brainbank.nl). In compliance with all local ethical and legal guidelines [36], informed consent for brain autopsy and the use of brain tissue and clinical information for scientific research was given by either the donor or the next of kin. The procedures of the Netherlands Brain Bank (Amsterdam, The Netherlands) were approved by the Institutional Review Board and Medical Ethical Board (METC) from the VU University Medical Center (VUmc), Amsterdam. Brains were dissected in compliance with standard operating protocols of the Netherlands Brain Bank and BrainNet Europe, after a 4-week fixation in 4% formaldehyde (more details on the full NBB autopsy and dissection procedure are published online: https://www.brainbank.nl/brain-tissue/autopsy/).

The details of all donors included in this study are listed in Online Resource Table 1. Most of these PD donors developed symptoms of dementia during their disease course and had extensive α-synuclein pathology throughout the brain (Braak LB stage 5/6) [6]. In addition, PD donors with earlier Braak stages (Braak LB stage 3/4) were included, as well as iLBD cases that did not develop clinical Parkinson’s disease but displayed Lewy pathology in their brain at autopsy (Braak LB stage 3), and controls (Braak LB stage 0) [6]. Formalin-fixed paraffin-embedded (FFPE) tissue blocks of the substantia nigra (SN) and hippocampus—also containing part of the parahippocampal gyrus- from these donors (details in Online Resource Table 1) were cut into 10 and 20 µm thick sections, which were utilized for immunohistochemistry and multiple labeling experiments. In addition, snap-frozen tissue blocks of the SN from 5 patients with advanced PD were cut into 10 µm for CARS microscopy (specified in Online Resource Table 1).

### Generation and detailed description of the characterization of novel aSyn-specific antibodies

#### Information provided in Online Resource Methods

##### Immunohistochemistry

**Fig. 1:**
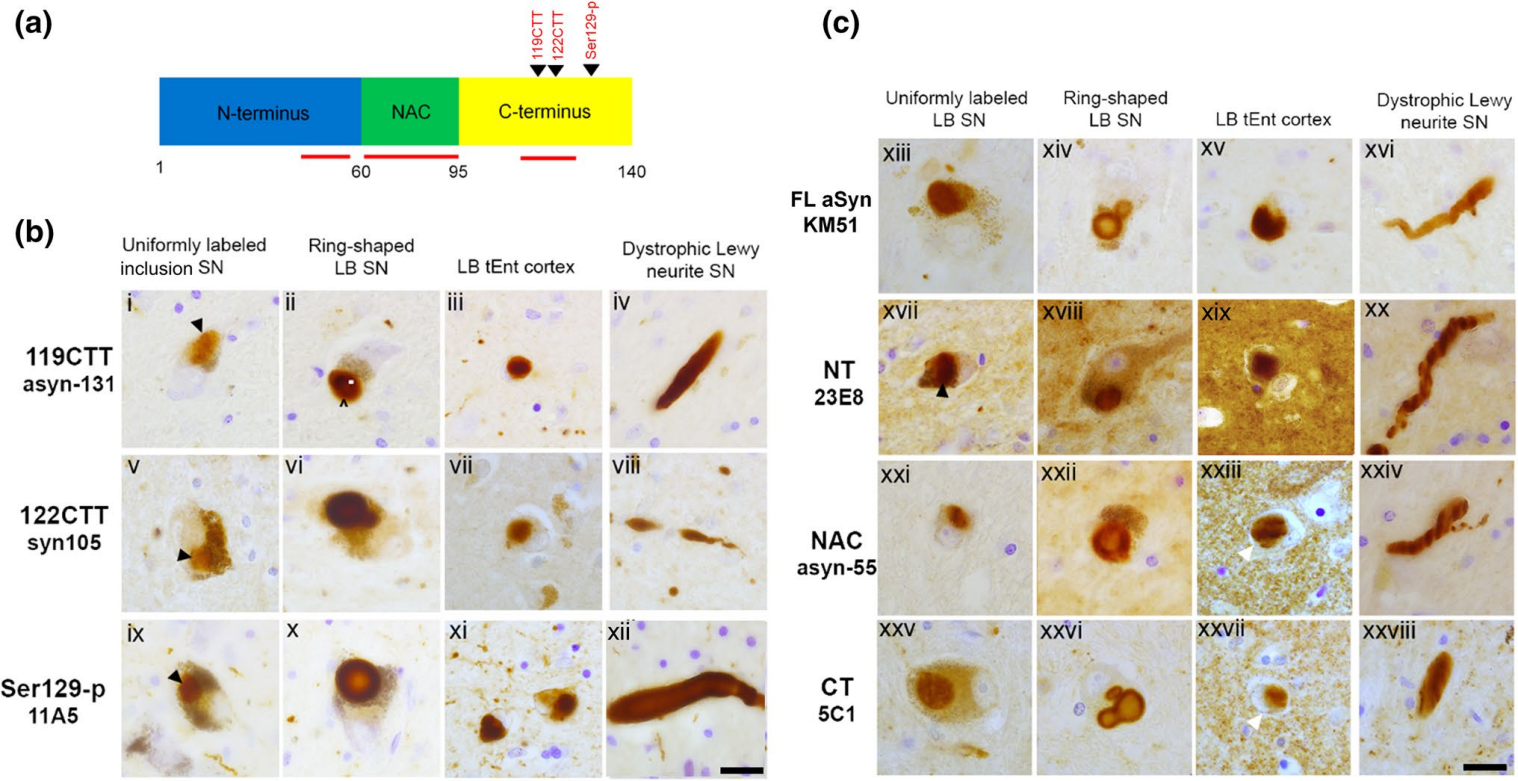
Characterization of IHC patterns for antibodies against different aSyn domains and PTMs in post-mortem brains with advanced PD pathology. **a** Schematic outline of the aSyn protein together with regions/PTM variants targeted by the antibodies applied in the present study (in red; summarized in Online Resource Table 2). **b/c** Representative brightfield images for selected morphological structures detected by antibodies against different aSyn PTMs (**b**) and domains (**c**). IHC labeling patterns of the antibody KM-51, an antibody commonly used for neuropathological diagnosis [2], are also included. All images were captured in the SN or transentorhinal cortex of the same advanced PD patient (Braak 6). Different IHC features are flagged (discussed in text). Black and white arrowheads highlight aSyn+ neuronal inclusions. Scale bar = 20 µm

Protocols for the antibodies against aSyn were optimized for light microscopy to characterize their immunoreactivity in human post-mortem formalin-fixed paraffin-embedded brain tissue. All IHC protocols could be optimized without antigen retrieval procedure and without addition of Triton. The EnvisionTM + kit (DAKO, Santa Clara, USA) was used as a high-sensitivity visualization system, with 3,3’-diaminobenzidine (DAB; 1:50 diluted in substrate buffer; DAKO) as the visible chromogen. Stained sections were analyzed using a Leica DM5000 B photo microscope (Leica Microsystems, Heidelberg, Germany). All brightfield images included in Fig. 1 were acquired using a HC PL APO 63 × 1.40 oil objective using a Leica DFC450 digital camera (Leica Microsystems).

### Development of multiple labeling protocols

#### Immunoreactivity patterns of aSyn epitopes

Using immunofluorescent stainings, antibodies against different domains and PTMs of aSyn were co-visualized and their local distribution patterns were assessed in pathological structures and within neuronal subcellular compartments. Double labeling experiments using two antibodies against aSyn were initially performed to obtain insight into their distribution patterns. Moreover, to allow systematic comparison of distribution patterns of different aSyn epitopes, protocols were developed to visualize multiple (4 or 5) antibodies against aSyn in the same section. To validate findings from these multiple labeling experiments, a ‘validation set’ of different antibodies against similar epitopes were selected and optimized for additional multiple labeling protocols. The sets of antibodies used in the different protocols are specified in Online Resource Table 2. No antigen retrieval methods or permeabilization steps were applied in any of these experiments.

For each protocol, we made use of a combination of direct and indirect immunodetection methods. Several primary antibodies (specified in Online Resource Table 2) were directly labeled with fluorochromes following standard protocols of different labeling kits (art. no. A20181, A20183, A20186, 21,335 for labeling with Alexa 488, Alexa 546, Alexa 647, and biotin, respectively; Thermo Fisher Scientific, Waltham, USA). Each protocol started with an indirect immunolabeling using unlabeled primary antibodies raised in rabbit/mouse using their appropriate secondary antibodies (with different conjugates, specified in Online Resource Table 2). Sections were then blocked for 1 h in 5% normal rabbit serum and 5% normal mouse serum in PBS. After this, a biotinylated primary antibody (raised in mouse or rabbit) could be incubated and visualized by fluophore-conjugated streptavidin. Then, sections were incubated in blocking solution (2% normal goat serum) containing the diluted directly labeled antibodies together with DAPI (1 µg/ml). Sections were mounted in Mowiol mounting solution using glass cover slips (Art. No.: 630–2746; Glaswarenfabrik Karl Hecht, Sondheim, Germany). Negative control stainings lacking primary antibodies were performed to control for background/autofluorescence levels and aspecific staining. Single stainings using a directly labeled antibody against Ser129-p aSyn were scanned to determine autofluorescence levels of the studied morphological structures (LBs, LNs), which was found negligible under the applied scan settings.

#### Association CTT and Ser129-p aSyn with subcellular markers

To study the association of immunoreactivity of CTT and Ser129-p aSyn with subcellular structures, additional multiple labeling protocols were further designed. Apart from the described antibodies against aSyn, these protocols also included some commercial antibodies as markers for subcellular structures, including mitochondria, ER and cytoskeletal proteins (Online Resource Table 2). In these protocols, heat-induced epitope retrieval using citrate buffer (pH 6.0) and a permeabilization step (1 h incubation in 0.1% Triton-x) was added to the protocols. Negative control stainings lacking primary antibodies were included to control for background/autofluorescence levels and aspecific staining.

### Confocal and STED microscopy

CSLM and STED microscopy were performed using a Leica TCS SP8 STED 3X microscope (Leica Microsystems). All images were acquired using a HC PL APO CS2 100 × 1.4 NA oil objective lens, with the resolution set to a pixel size of 20 nm × 20 nm. All signals were detected using gated hybrid detectors in counting mode. Sections were sequentially scanned for each fluorophore, by irradiation with a pulsed white light laser at different wavelengths (indicated in Online Resource Table 2). Stacks in the Z-direction were made for each image. To obtain CSLM images of the DAPI signal, sections were irradiated with a solid state laser at a wavelength of 405 nm. For STED imaging, a pulsed STED laser line at a wavelength of 775 nm was used to deplete Abberior (580, 635P), Alexa (594, 647) or Li-Cor (680 nm) fluorophores, while continuous wave (CW) STED lasers with wavelengths of 660 nm and 592 nm were used to deplete the Alexa 546 and Alexa 488 fluorophores, respectively. The DAPI signal was not depleted, so this channel was scanned at the same resolution as the CSLM images.

After scanning, deconvolution was performed using CMLE (for CSLM images) and GMLE algorithms (for STED images) in Huygens Professional (Scientific Volume Imaging; Huygens, The Netherlands) software. Images were adjusted for brightness/contrast in ImageJ (National Institute of Health, USA). 3D reconstructions were made using the LAS X 3D Visualization package (Leica Microsystems). Final figures were composed using Adobe Photoshop (CS6, Adobe Systems Incorporated).

For the comparison of different Ser129-p aSyn+ profiles in iLBD versus advanced PD, midbrain sections of controls (*N* = 3), iLBD Braak 3/4 donors (*N* = 6) and Braak 6 PD patients (*N* = 3) were labeled for Ser129-p aSyn (asyn-142), neurofilament, and TH. Transmitted light scans were made for the identification of neuromelanin-containing neurons. 3D Tile scans (z-stack = 3 µm) were acquired and merged on a Leica TCS SP8 STED 3X microscope (Leica Microsystems) using LASX Navigator software (Leica Microsystems). Scans were made using a HC PL APO CS2 63 × 1.4 NA oil objective lens with a zoom factor of 1.0 at 1024 × 1024 pixels. All signals were detected using gated hybrid detectors in counting mode.

### Image processing and (semi-)quantitative analyses

Nigral LBs were classified and selected for inclusion in the analysis based on their immunopositivity for Ser129-p in combination with morphological criteria (specified in Results section). Additional criteria for inclusion were 1) the diameter of the structure (at least 5 µm) and 2) the presence of specific signal for all channels (signal intensity of raw CSLM images substantially higher than autofluorescence or background levels under the applied scan settings). In this selected subset of LBs, distribution patterns of immunoreactivities were analyzed on deconvolved CSLM images of 30 LBs in the SN of 8 patients with advanced PD (Online Resource Table 1). Z-stacks were made for each structure, of which three frames in the central portion of the structure (Z length: 0.30 µm; step size between frames: 0.15 µm) were selected to quantify the x–y distribution for different markers. For the analysis, a maximum Z-projection of these selected frames was first made in ImageJ. Subsequently, three 100 px (2 µm) thick lines were drawn over three equatorial planes of the LBs (similar to [22]) in ImageJ, along which signal intensities for each channel were measured using a custom-made script. The average intensity for each channel at each point of the diameter was normalized to its maximum intensity in the same structure, while the position along the diameter was expressed as % diameter. Normalized values were used to generate average line profiles per morphological structure. The center of the LB was defined as the origin of the structure [22]. The position in the LB with the maximum intensity was determined per channel. Ranking of absolute positions of maximum intensities per structure with respect to the origin of the LB were compared between channels (nonparametric Friedman test). P-values for multiple comparisons were adjusted using Dunn’s correction for multiple comparisons.

For the quantification of different Ser129-p aSyn+ morphologies in iLBD Braak 3/4 donors and PD Braak 6 patients, a maximum projection of 3D merged tile scans of the SNpc was made in ImageJ. Dopaminergic neurons were identified by neuromelanin-content in brightfield scans and confirmed by TH immunoreactivity. Neuromelanin-containing neurons were identified and counted using thresholding by an ImageJ algorithm (Otsu), and the ROI area was measured. After this, each individual neuron was assessed for the presence of somatic Ser129-p aSyn+ profiles, which were manually classified by an assessor that was blinded for the donor’s diagnosis. We distinguished between Ser129-p aSyn+ cytoplasmic network profiles in absence of apparent LB-like inclusions/Ser129-p aSyn+ uniform inclusions with or without cytoplasmic network profiles/Ser129-p aSyn+ ring-shaped inclusions with or without cytoplasmic network profiles. The relative proportion of specific subcellular profiles to the total pool of Ser129-p aSyn+ cells was compared between groups of iLBD and PD donors. For inclusions (> 5um) with uniform and ring-shaped appearances, their association with a cytoplasmic Ser129-p aSyn+ network and with neurofilament+ profiles were also studied. The number of neuromelanin-containing neurons and the percentage of Ser129-p aSyn+ cells were compared between iLBD and PD patients using Student T Tests.

Statistical analyses were done using SPSS software (version 22, IBM) and GraphPad software (version 7.0, Prism).

### Coherent anti-Stokes Raman scattering

The workflow used for CARS microscopy is outlined in Online Resource Fig. 9. The detection of the lipid and protein distribution was performed on native, dried samples [16, 60]. A commercial setup (Leica TCS SP5 II CARS, Leica Microsystems) was used with an HCX IRAPO L25X/0.95 W (Leica Microsystems) objective. For the lipid distribution intensity images were taken at 2850 cm^−1^ (Pump-wavelength 816 nm, Stokes-wavelength 1064 nm) and for the protein distribution intensity images at 2930 cm^−1^ (Pump-wavelength 810 nm, Stokes-wavelength 1064 nm). The laser power at the sample was 28 mW (Pump) and 21 mW (Stokes). Integration times of 34 s per image with a pixel dwell time of 32 µs, 1024 × 1024 pixels and a spatial resolution of 300 nm were used [54]. After the label-free detection of the lipid and protein distribution, immunofluorescent stainings were performed on the same sections (Online Resource Fig. 9). Tissue sections were fixed in 4% formaldehyde for 10 min and stained for aSyn, using two primary antibodies raised against aSyn (LB509; ab27766, Abcam, Cambridge, UK) and Ser129-p aSyn (ab59264, Abcam) and their appropriate secondary antibodies. After this, sections were incubated in Sudan Black for 30 min and mounted in Mowiol. For fluorescence detection, a commercial setup (Leica TCS SP5 II CARS, Leica Microsystems, Heidelberg, Germany) was used. Data evaluation was done in Matlab with the Image Processing and Statistics toolboxes (The Mathworks, Inc., Mass.,USA). First, large overview CARS intensity and fluorescence images were manually overlaid by comparison of morphological features. The distribution of aSyn, proteins and lipids were identified by the overlay of both fluorescence images (Online Resource Fig. 9). Therewith, autofluorescence of the surrounding tissue and the fluorescent signal of the aSyn+ inclusions could be separated. The inclusion bodies were manually identified based on morphology. Only inclusions with a diameter of 5–20 µm were included for analysis.

### Image processing and analysis of CARS images

For an objective evaluation of CARS intensity of aSyn+ inclusion bodies, the mean CARS intensity of the direct surrounding, a donut with a width of 3.5 µm, was compared with the CARS intensity of the inclusion (Online Resource Fig. 10, light blue and yellow area). The areas of aSyn-immunopositivity were transferred into the CARS-intensity images. Areas with no intensity in the CARS-intensity images (holes) were excluded by intensity thresholding. CARS-pixel-intensities higher than 1.4 times the mean CARS intensity of the surrounding were defined as higher protein/lipid content, which was determined based on pilot measurements in a subset of (~ 40) aSyn-positive inclusions. The ratio between the CARS-pixel-intensities of the LB and the mean CARS intensity of the surrounding were calculated and the areas with higher protein/lipid content were marked in red (Online Resource Fig. 10). Morphological filtering and image processing were performed in Matlab R2017a, MathWorks.

## Results

### Epitope-specific antibodies against aSyn proteoforms capture its various manifestations in the human brain

To study the subcellular distribution patterns of different aSyn proteoforms, we compiled a set of epitope-specific antibodies against CTT and Ser129-p aSyn and different domains of the protein (N-terminal, NAC region, C-terminal). A summary of all antibodies used in this study is provided in Online Resource Table 2. To replicate initial results on the localization patterns of CTT and Ser129-p aSyn in human brain tissue [62], syn105 and 11A5 antibodies were included. The initial characterization and validation of these antibodies has been described in literature [3, 23, 24] Syn105 is an antibody raised against an immunogenic peptide corresponding with residues 118–122 of aSyn, which displays a high affinity for 122CTT aSyn fragments compared to full-length aSyn [23], while 11A5 is directed specifically against aSyn phosphorylated at Ser129 [3]. As expected based on their reported epitopes, 11A5 and syn105 detected Ser129-p aSyn or 122CTT aSyn using WB on recombinant proteins, respectively, and displayed lack of binding to other forms of aSyn (e.g. full-length aSyn; 119CTT aSyn; Online Resource Fig. 1b).

To further validate results obtained using the 11A5 and syn105 antibodies, we employed an additional set of novel monoclonal antibodies directed against 119CTT (asyn-131), 122CTT (asyn-134), and Ser129-p aSyn (asyn-142; initially published as 7E2 [1]). The specificity of these novel antibodies was confirmed using enzyme-linked immunosorbence assays (ELISAs) and surface plasmon resonance (SPR) on aSyn peptides as well as by Western blots (WB) on recombinant protein (details provided in Online Resource Methods, Online Resource Fig. 1 and Online Resource Tables 3 and 6). All immunostaining results for Ser129-p and 122CTT aSyn presented in this study were cross-validated using different antibodies.

As comparison for the observed immunoreactivity patterns for Ser129-p and CTT aSyn, we included antibodies directed against epitopes localized at specific domains of aSyn. To probe for aSyn’s CT, we included two antibodies with a similar epitope for which the characterization and epitope mapping were previously described in literature: 5C1 (res. 118–125) and 211 (res. 121–125) [23, 24, 26]. Based on their epitopes, such antibodies are expected to detect recombinant full-length and Ser129-p aSyn but not 119CTT and 122CTT, which was confirmed by WB on recombinant proteins (Online Resource Fig. 1b). In contrast, an antibody with an epitope at res. 40–55 of aSyn’s NT (23E8) also showed as expected immunoreactivity to recombinant CTT, full-length and Ser129-p aSyn by WB (Online Resource Fig. 1b). Lastly, two antibodies (asyn-055 & asyn-058) were included in our analyses that showed strong binding to full-length, recombinant aSyn protein in ELISAs but not to its NT (res. 1–60) or CT (res. 96–140) protein fragments (Online Resource Table 3). Based on this result, binding for these antibodies to a conformational epitope in the NAC domain was presumed. In support of this, WB on recombinant proteins showed detection of full-length, CTT aSyn, and Ser129-p aSyn, similar to 23E8 (Online Resource Fig. 1b). The results of this antibody characterization using WB on recombinant proteins are summarized in Online Resource Table 3.

To characterize antibody binding in human tissue, an exploratory WB analysis was performed on putamen samples from two controls and two advanced DLB patients (Braak 6) by extracting the tissue in 4-(2-hydroxyethyl)-1-piperazineethanesulfonic acid (HEPES)/sucrose buffer. After separating the extract by centrifugation into a detergent-free soluble and an insoluble fraction, we performed a gel electrophoresis and examined the immunoreactivity pattern of selected monoclonal antibodies to proteins extracted from the post-mortem brain tissue by WB. Since the extraction method is devoid of any detergents, the insoluble fraction may contain aSyn bound to lipids or other water insoluble proteoforms of aSyn. In both soluble and insoluble fractions, antibodies against NT, NAC and CT aSyn epitopes all showed bands at 14 kDa, corresponding with monomeric full-length aSyn (Online Resource Fig. 1c) in DLB patients and controls. In line with the WB results that were obtained using recombinant proteins, antibodies against NT and NAC aSyn also detected lower molecular weight (MW) fragments—both in DLB patients and controls—corresponding with truncated aSyn species (Online Resource Fig. 1c). This supports previous findings that truncated aSyn fragments are present not only in situations of pathology but also under physiological conditions [44, 52]. Bands below 14 kDa were not observed for antibodies against CT aSyn, indicating that such aSyn fragments are mainly CTT. Additionally, antibodies against NT/NAC aSyn (and to a lesser extent against CT aSyn) detected variable bands between 14 and 62 kDa in HEPES/sucrose-soluble and insoluble fractions of DLB patients and controls (Online Resource Fig. 1c), possibly reflecting the presence of multimeric aSyn variants or aSyn assemblies that are sodium dodecyl sulfate (SDS) stable.

Ser129-p aSyn was detected exclusively in HEPES/sucrose-insoluble fractions of DLB patients, but not controls, consistent with previous observations that insoluble Ser129-p aSyn is enriched under pathological conditions (Online Resource Fig. 1c) [3, 21, 27]. Both monomeric and a spectrum of insoluble higher MW species were detected, including signal in the gel loading pockets reflecting large aSyn assemblies that are SDS stable. Similarly, insoluble high MW features in gel loading pockets of DLB patients but not controls were detected using antibodies against 119CTT and 122CTT aSyn in DLB patients, supporting that incorporation of CTT aSyn in insoluble, high MW complexes is associated with pathology [5]. However, the spectrum of other disease-associated insoluble monomeric and higher MW fragments as seen in the Ser129-p aSyn WB were less prominent using CTT antibody. After heat-induced antigen retrieval of the WB membrane, bands for aSyn species were observed at MW lower than 14 kDa using antibodies against CTT aSyn in HEPES/sucrose-soluble (Online Resource Fig. 1d) and insoluble fractions (not shown) of both patients and controls, but not at 14 kDa. This result is consistent with the detection of shortened aSyn proteoforms rather than full-length aSyn as observed by WB on recombinant protein. Additional bands using antibodies against 122CTT and (to lesser extent) 119CTT were observed at molecular weights higher than for monomeric aSyn (e.g. around 55 kDa) both in patients and controls, suggesting that such potential multimeric species or assemblies are formed in the brain also under conditions of normal aging. Our main findings of the WB analysis on human brain-tissue specimens using the selected antibodies are summarized in Online Resource Table 4.

Based on our initial characterization of the selected antibodies using biochemical techniques, we confirm their detection of the targeted epitopes in peptide assays, with recombinant proteins, and in DLB patients and normal aged brains, revealing various manifestations of aSyn under physiological and pathological conditions.

### Profiles of Lewy pathology as detected by different aSyn antibodies

For the assessment of subcellular distributions of the different aSyn proteoforms in multiple labeling protocols, we first developed immunohistochemistry protocols for all selected antibodies on FFPE tissue sections in the absence of additional antigen retrieval methods (i.e. no proteinase K digestion was applied). Representative images of neuronal inclusions labeled by antibodies against different aSyn epitopes and proteoforms—taken in the SN, hippocampus and transentorhinal cortex of PD patients—are shown in Fig. 1, together with KM-51, which is an antibody commonly used for neuropathological diagnosis according to established protocols including formic acid pretreament and heat-induced antigen retrieval in citrate buffer [2].

In line with existing literature [3, 27, 62], immunohistochemical (IHC) stainings using antibodies directed against CTT aSyn species (119 and 122 CTT aSyn) and Ser129-p aSyn showed labeling of a variety of pathology-associated morphologies in the PD brain, including neuronal cytoplasmic inclusions such as LBs and LNs. Such morphologies were also detected by antibodies directed against epitopes within specific domains (CT, NT and NAC domain) but in the presence of prominent staining of the neuropil (most strongly in the hippocampus and transentorhinal cortex) while such patterns were observed to far lesser extent for PTM-specific antibodies (Fig. 1b, xix, xxiii, xxvii). These features are consistent with synaptic-like staining profiles [39]. Their presence in patients as well as controls indicates that antibodies against CT, NT and mid-region aSyn domains detect physiological aSyn. In contrast, immunoreactivity for CTT and Ser129-p aSyn was mainly restricted to pathology-associated structures in PD patients, which is in support of relative enrichment of these variants in pathological inclusions [21].

aSyn+ neuronal inclusions in PD have been described to exhibit substantial morphological heterogeneity, amongst others determined by inclusion size, brain region, and specific cell type [70]. Based on immunostaining patterns for different aSyn antibodies in different brain regions (SN, hippocampus, transentorhinal cortex), we distinguished between two major types of neuronal somatic inclusions. First, in a subset of spherical LBs (mainly observed in the SN), immunoreactivity for aSyn antibodies revealed a ring-shaped appearance, i.e. with an immunopositive band surrounding a central—weakly or unstained—core (Fig. 1b/c). The ring-shaped appearance of midbrain LBs has been described using antibodies against aSyn in literature [62], and a subset of these morphologies was shown to represent eosinophilic ‘classical LBs’ unambiguously identified by hematoxylin and eosin (H&E) stainings [40]. Inspection in adjacent brain sections of the same patients suggested that this ring-shaped appearance of LBs was most clearly visualized by antibodies against Ser129-p aSyn and CT aSyn (Fig. 1b/c, x, xxvi), while antibodies directed against other aSyn epitopes generally revealed less contrast between core and immunoreactive ring. For antibodies against CTT aSyn, an area of weaker immunoreactivity surrounding the strongest immunopositive portion of LBs could occasionally be observed (e.g. Fig. 1a, ii). Although these observations suggest that antibodies against aSyn proteoforms highlight different organizational aspects of LBs, confirmation using double and multiple immunolabelings is required to confirm this observation (discussed in the next paragraphs). Staining patterns revealing peripheral immunoreactivity surrounding a weakly labeled central core could also be observed in certain dystrophic LNs in the SN (Fig. 1b, xii).

Other neuronal aSyn+ inclusion bodies revealed a more diffuse and uniform labeling throughout the structure for all tested antibodies. This IHC pattern was generally observed for compact limbic and cortical LBs in the hippocampal CA2 region and transentorhinal cortex, respectively, but also in the SN [40, 70]. In the SN, a subset of inclusions with uniform aSyn labeling probably represent pale-body like structures which are defined based on a relative lack of reactivity in H&E stainings compared to classical LBs but can be strongly immunoreactive for Ser129-p aSyn [37, 40, 70]. Based on the labeling patterns by different aSyn antibodies, we did not observe major differences between cortical LBs and nigral inclusions without ring-shape appearance. The size and shape of such inclusions uniformly stained for aSyn showed substantial heterogeneity, including compact globular and irregularly-shaped, expansive-appearing cytoplasmic inclusions, as has been previously described [34, 40, 70]. In the SN, cells were commonly found to contain multiple inclusion bodies. Both combinations of multiple ring-shaped inclusions, multiple uniformly labeled inclusions, as well as combinations of ring-shaped inclusions and uniformly stained inclusions were observed within the same cells in vicinity or even in continuum. This observation was done using different aSyn antibodies and is in accordance with previously published observations [70].

In summary, the selected antibodies against aSyn proteoforms and domains in our study all showed detection of various neuronal PD-relevant pathological structures in different brain regions, while prominent synaptic-like staining of the neuropil was only observed for antibodies against aSyn domains in patients and controls.

### A subset of LBs displays a consistent onion skin-like organization

To compare the distribution patterns of aSyn epitopes in the same LB and LN morphologies in more detail, immunofluorescent multiple labelings were performed using different combinations of antibodies against aSyn proteoforms and domains. Labeled features were inspected by high-resolution 3D multicolor confocal and STED microscopy. As discussed in the previous section a gross distinction between two types of inclusions was made based on either ring-shaped or uniform aSyn labeling patterns during initial brightfield immunohistochemical characterizations. Particularly antibodies against Ser129-p aSyn allowed sensitive and consistent detection of and separation between these inclusion morphologies. In the different analyses performed in this study, we did not observe LBs without labeling for Ser129-p aSyn that were immunopositive for other epitopes, while the opposite (immunolabeling for Ser129-p aSyn but not for other epitopes) was occasionally observed.

**Fig. 2:**
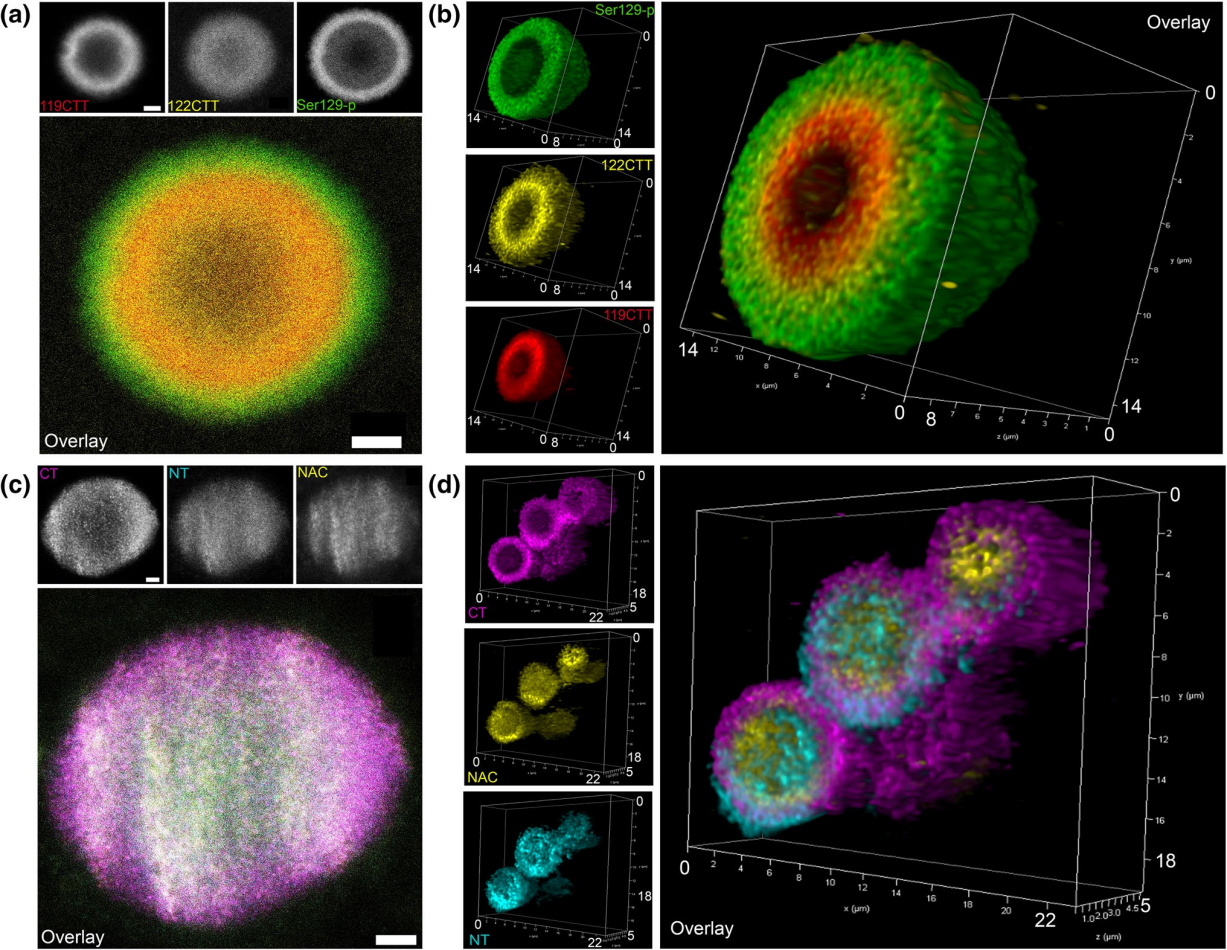
Differential localization of antibodies directed against aSyn PTMs and aSyn domains in LBs. Antibodies shown: 119CTT: syn-131, 122CTT: syn105, Ser129-p aSyn: 11A5. **a** Triple labeling of aSyn PTMs: representative raw STED image of a nigral LB in a PD patient, showing immunoreactivity for Ser129-p aSyn at the periphery of the LB while 119CTT (asyn-131) and 122 CTT aSyn are localized in the core of the structure. **b** 3D reconstruction based on deconvolved CSLM images showing a lamellar distribution of different aSyn PTMs. **c** Triple labeling of antibodies directed against different aSyn domains (CT/NT/NAC domain): raw STED image of a nigral LB, showing immunoreactivity for CT aSyn at the periphery of the LB and NT and NAC aSyn staining in the core of the structure. **d** 3D reconstruction based on deconvolved CSLM images showing different localization for aSyn domains in nigral LBs. Scale bar in **a** and **c**: = 2 µm

When analyzing the subset of ring-shaped LBs in the SN using different combinations of antibodies (Online Resource Table 2), we observed only partial co-localizion for Ser129-p aSyn, 119CTT and 122CTT. In particular, regions within a LB immunoreactive for Ser129-p in nigral LBs localized consistently more towards the periphery compared to 119CTT and 122CTT aSyn, which were found condensed in the LB core (Fig. 2a, c, Online Resource Video 1,2). Thereby, we reproduced the results described in[62]. Interestingly, similar different distributions patterns were also observed between antibodies against NAC, NT and CT domains of aSyn, with CT aSyn immunoreactivity at the periphery of the other domains (Fig. 2b, d, Online Resource Video 3). As the antibodies against CT aSyn did not recognize CTT aSyn in WB experiments on purified proteins or in tissue, in contrast to NT/NAC antibodies (Online Resource Fig. 1b,c), these observations further indicated a separation between aSyn proteoforms with intact versus truncated CT domains in this subset of LBs.

**Fig. 3:**
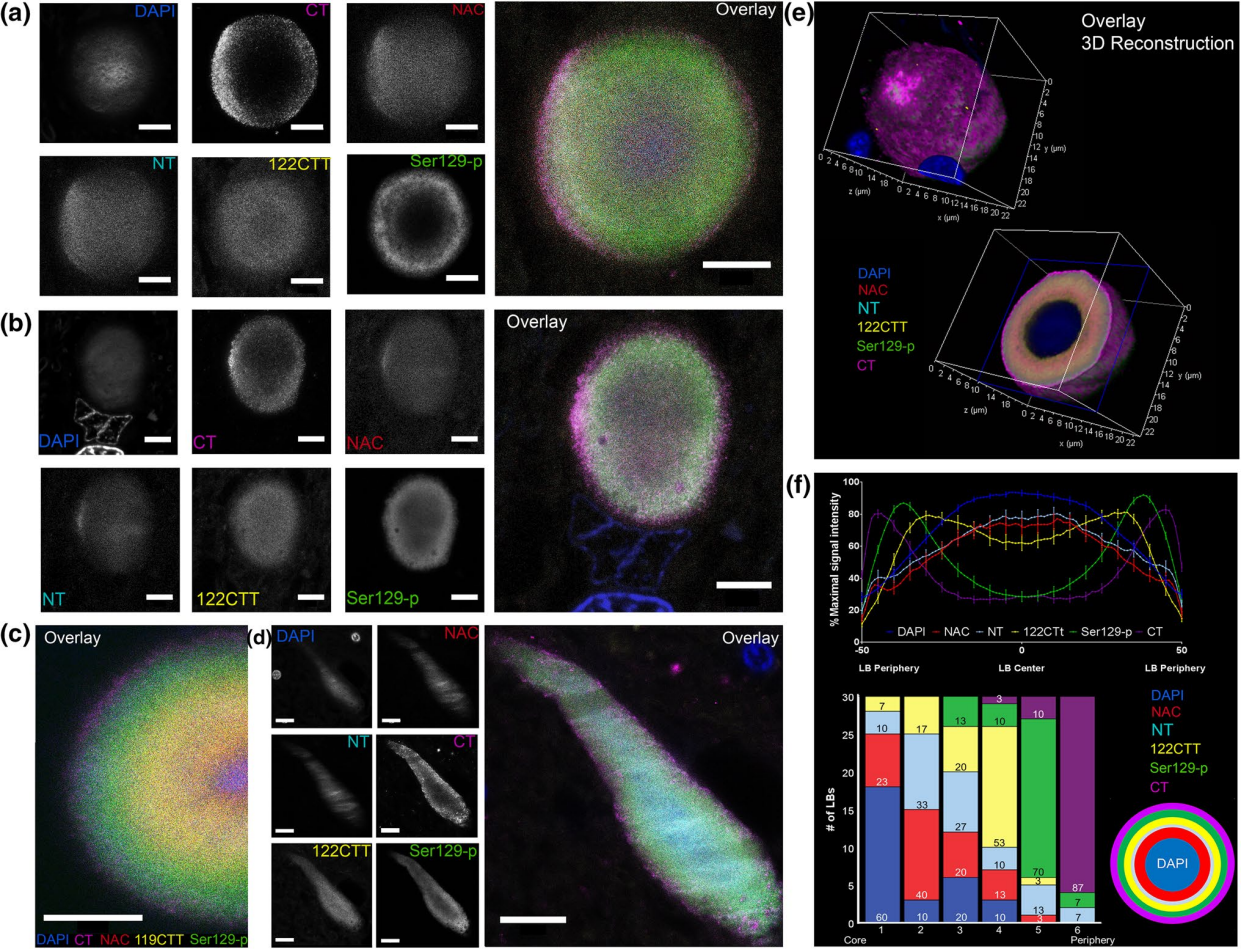
Different aSyn epitopes highlight a consistent onion skin-like arrangement of different aSyn epitopes in nigral LBs and LNs. Antibodies used in Figure/analyses: CT: 5C1; NT: 23E8; NAC: asyn-55; 122CTT: syn105; 119CTT: asyn-131; Ser129-p aSyn: 11A5. **a, b** Raw STED images showing immunoreactivities for different aSyn epitopes in onion skin-type LBs in the SN of patient PD1 (**a)** and PD5 (**b**). Immunoreactivities for CT and Ser129-p aSyn are localized at the periphery of the structures, while NT, NAC and 122CTT reactivities were present mainly in their core. **c** Raw STED image of multiple labeling including an antibody against 119CTT aSyn, taken in the SN of patient PD5. **d** Raw STED image of a dystrophic LN in the SN of patient PD7. **e** 3D reconstruction of an entire nigral LB based on deconvolved CSLM images. **f** Top: Average line profile (± SEM) for 30 onion skin-like LBs measured in the SN of 8 PD patients, showing a separation of peak intensities. Bottom: summary of rankings of peak intensity locations from core to periphery for the studied aSyn epitopes, highlighting peripheral localization of CT and Ser129-p aSyn. Right: a schematic depiction of the lamellar architecture of LBs as revealed by antibodies against different aSyn epitopes. **a-c** Scale bar = 5 µm; **d**: Scale bar = 10 µm

When combining antibodies against different epitope groups in multiple labeling protocols, the gradual and distinct distribution of immunoreactivities in ring-shaped nigral LBs became more evident (Fig. 3a). The lamellar organization of different concentric rings together demonstrated an onion skin-like morphology, composed of layers enriched for different aSyn proteoforms. CT and Ser129-p aSyn showed immunoreactivity at the periphery of LBs and very limited labeling of their core, while antibodies against CTT aSyn, NT and NAC aSyn collectively clustered more in the central portion of LBs (Fig. 3a–c). This result was reproduced using a set of different antibodies directed against similar epitopes (Online Resource Fig. 2; Online Resource Table 2), while the localization for antibodies against different CTT aSyn variants (119CTT and 122CTT) in the LB core was similar (Fig. 3c). 3D CLSM analyses showed the lamellar organization of this subset of LBs throughout the entire structure (Fig. 3e, Online Resource Video 4). Interestingly, certain dystrophic LNs in the SN were observed to contain similar lamellar compositions (Fig. 3d). Converging fluorescence for DAPI (a DNA binder), was consistently observed at the core of onion skin-type LBs and LNs, although this signal was generally substantially weaker than its staining intensity in cell nuclei (Fig. 3b).

To test the consistency of the onion skin-like structure of LBs, we semi-quantitatively examined 30 LBs in formalin-fixed paraffin-embedded SN sections from 7 PD patients (Online Resource Table 5). LBs included for analysis displayed a ring-shaped appearance, were localized in the cytoplasm of neuromelanin-containing dopaminergic neurons and had a diameter larger than 5 µm (inclusion criteria are further explained in *Material and Methods* section). For each of the scanned LBs, relative signal intensities were plotted per channel over a normalized LB diameter to generate line profiles that visualize the distributions of aSyn epitopes in each LB. The average line profile over the 30 LBs showed a clear separation of peak intensity localizations for the different aSyn epitopes, demonstrating their consistent distribution in the LBs selected for analysis (Fig. 3f, upper panel). In addition, we determined the position in each LB where the peak intensity for each aSyn epitope was localized relative to the LB origin, which we ranked among the different epitopes (Fig. 3f, lower panel). A different distribution of peak intensities was confirmed by statistical analysis of this data (*χ*^2^: 73.912 (4); *p* < 0.0001; Fig. 3f; post-hoc tests presented in Online Resource Table 6).

Peak intensities of 122CTT aSyn were localized at a more central position compared to Ser129-p aSyn in almost all (97%; Online Resource Table 5) analyzed LBs. Moreover, immunoreactivity of 122CTT aSyn was localized more to the core of LBs than antibodies against res 118–126 of aSyn’s CT in 97% of the analyzed LBs (Online Resource Table 5, Fig. 3f). This finding shows that the antibodies against 122CTT and CT aSyn in our study recognize different aSyn proteoforms in human tissue and indicate that most of aSyn with an intact or accessible 118–126 portion of the epitope aSyn is present at the extreme periphery of LBs, while a substantial portion of aSyn in the LB core contains shortened CT (or that the epitope for the CT antibodies is masked). This effect could be more pronounced for truncations at the CT, as peak intensities for NAC and NT aSyn—which detect CTT aSyn fragments (Supplementary Fig. 1)—were found more towards the central portion of the LB (Fig. 3f). No differences in this organization of onion skin-type LBs were observed between the different patients included in our analysis (Online Resource Table 4).

Distribution patterns for different aSyn epitopes were also analyzed in neuronal inclusions without ring-shaped appearance, in the same sections (in case of SN) and in other sections of the same patients (hippocampus/transentorhinal cortex). Although unambiguously detected and outlined by uniform Ser129-p aSyn immunolabeling—with exception of typical ‘vacuolar-like structures’ lacking Ser129-p aSyn reactivity [8, 67]—these inclusions often revealed weaker signal intensities for the other epitopes (particularly 122CTT, NAC and NT aSyn) that were diffusely distributed throughout the inclusion (Online Resource Fig. 3a,d). In such inclusions, DAPI signal was barely increased compared to the surrounding. Overall, these heterogeneous and often irregularly-shaped inclusions appeared relatively unstructured—e.g. no systematic differences in distribution were observed for aSyn epitopes or other tested markers (e.g.. Online Resource Fig. 3a, Online Resource Video 5). We did not observe differences in the distribution patterns of different aSyn epitopes in cortical versus nigral inclusions without ring-shape. We have summarized the main observations in our multiple labeling experiments in the selected LB morphologies in Table 1. Taken together, our results show a morphology-dependent, consistent and structured-appearing onion skin-like arrangement of aSyn epitopes in the subset of ring-shaped LBs and LNs, while other inclusions (= without ring-shape) appear more heterogenous and unstructured.

**Table 1:** Summary of immunoreactive profiles for aSyn proteoforms and selected subcellular markers in different LB morphologies and surrounding neuronal cytoplasm as observed in multiple labelings

		aSyn epitopes								
		Ser129-p	119CTT	122CTT	NT	NAC	CT	DAPI	NF	bTub
Perinuclear aSyn+ inclusion bodies (PD patients)	Onion skin-type; periphery	+ +	−	−	±	±	+ +	−	+ +	+ +
	Onion skin-type; core	±	+ +	+ +	+ +	+ +	−	+ +	−	−
	Uniform; SN	+ +	+ +	+	+	+	+	±	±	±
	Uniform; Hip/TEC	+ +	+ +	+	+	+	+	±	±	±
Neuritic aSyn+ inclusions (PD patients)	Onion skin-type; periphery	+ +	±	±	±	±	+ +	−	±	±
	Onion skin-type; core	±	+ +	+ +	+ +	+ +	±	+ +	-	−-
	Uniform SN	+ +	+ +	+	+	+	+	±	±	±
	Uniform Hip/TEC	+ +	+ +	+	+	+	+	±	±	±
Synaptic-like labeling (PD patients & controls)	SN	−	−	−	+ +	+ +	+ +	−	−	−
	Hip/TEC	−	−	−	+ +	+ +	+ +	−	−	−
Cytoplasmic labeling (PD patients)	SN	+ +	±	+ +	−	−	−	−	+ +	+ +
	Hip/TEC	+ +	±	+ +	−	−	−	−	+ +	+ +
Cytoplasmic labeling (controls)	SN	−	±	+ +	−	−	−	−	+ +	+ +
	Hip/TEC	−	±	+ +	−	−	−	−	+ +	+ +

−: no immunolabeling; ± : weak labeling/barely enriched compared to surrounding (e.g. cytoplasm); + : unambiguous labeling of structure; +  + : strong labeling; Abbreviations: Hip: hippocampus, TEC: transentorhinal cortex

### Onion skin-type LBs contain a cytoskeletal framework associated with Ser129-p aSyn

**Fig. 4:**
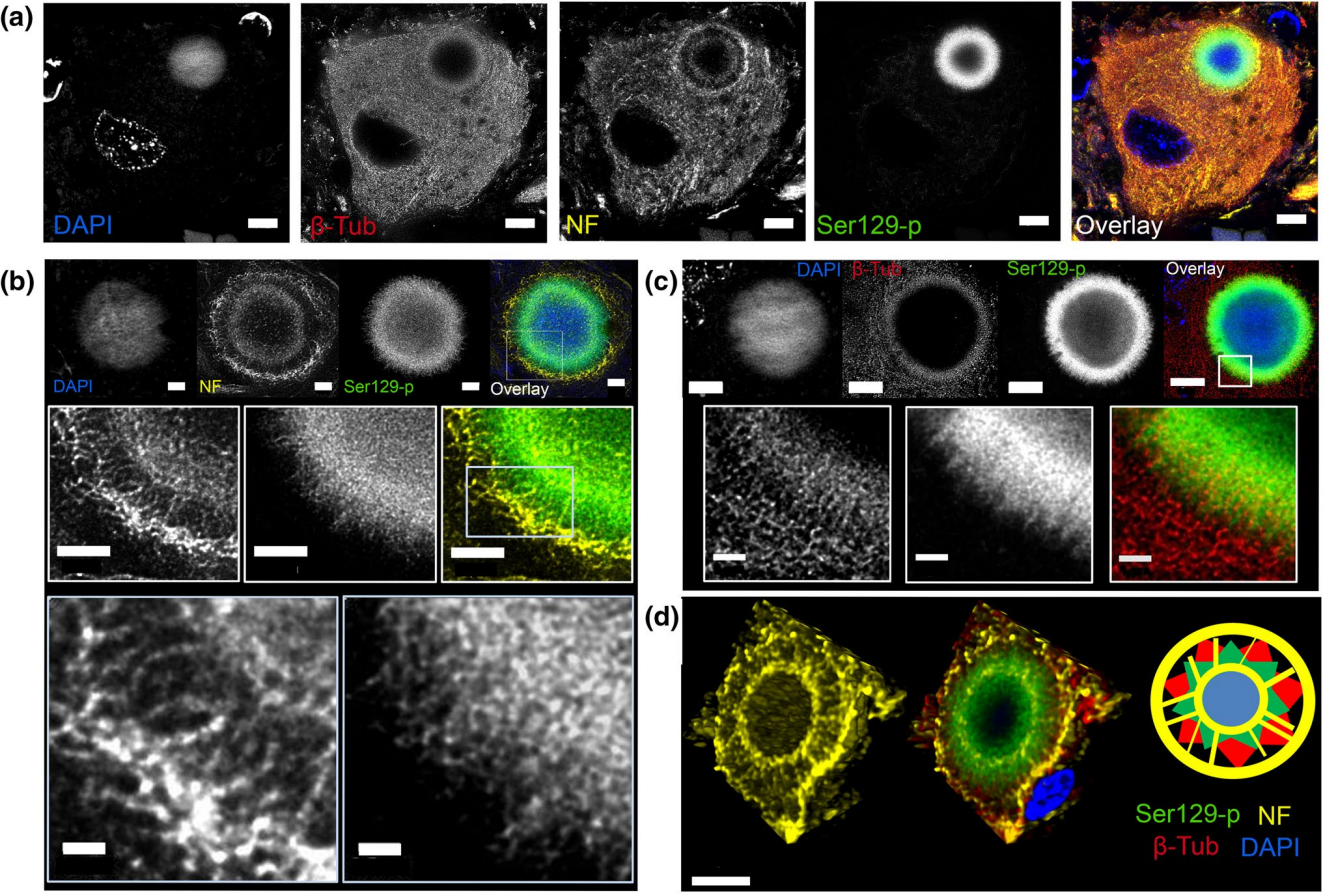
Ser129-p aSyn forms an intricate cage-like framework with cytoskeletal components at the periphery of nigral LBs. Ser129-p aSyn antibody for all images shown: asyn-142. **a** Deconvolved STED image of neuromelanin-containing dopaminergic neuron in the SN with LB. Immunoreactivity for beta-tubulin and neurofilament is observed at the periphery of the LB. **b** Deconvolved STED images showing the detailed structure of neurofilament in an onion skin-type LB at different magnifications. **c** Deconvolved STED images showing detailed beta-tubulin reactivity at the periphery of a LB. **d** Left: 3D reconstruction of the localization of Ser129-p aSyn and cytoskeletal components in a nigral LB, highlighting a wheel-like structure of neurofilament. Right: schematic summary of the results. NF: neurofilament; β-Tub: beta-tubulin. **a**: Scale bar = 5 µm; **b**: upper and middle row: scale bar = 2 µm; lower row: scale bar = 0.5 µm; **c**: Upper row: Scale bar = 5 µm, lower row: scale bar = 1 µm. **d**: Scale bar = 5 µm

The consistent arrangement of aSyn proteoforms in a subset of nigral LBs suggest that their morphogenesis may be extensively regulated. Major constituents in organizing cellular organelles and substructures are cytoskeletal proteins, for which immunoreactivity in LBs was described before [33, 41]. To obtain more insight into detailed localization for cytoskeletal components in onion skin-type LBs, we studied the immunoreactivity patterns of intermediate neurofilaments and beta-tubulin in these morphologies by 3D multicolor STED microscopy. Antibodies against intermediate neurofilament and beta-tubulin showed immunoreactivity mainly at the periphery of nigral onion skin-type LBs -without labeling of the LB core (Fig. 4)—in which their immunoreactive profiles outlined a structured-appearing peripheral framework intimately associated with the Ser129-p aSyn immunoreactivity.

Although LBs are generally observed in brightfield microscopy as spherical smooth-edged inclusions, detailed inspection revealed that the outline of many LBs revealed irregular and radiating Ser129-p aSyn immunoreactivity patterns (Online Resource Fig. 4). Beta-tubulin immunoreactivity showed substantial overlap with such radiating Ser129-p aSyn profiles at the LB periphery, although localized slightly more towards the outer LB portion (Fig. 4b). Antibodies against intermediate neurofilaments, in contrast, demonstrated a distinctive organization at the periphery of onion skin-type LBs. In particular, two immunopositive rings were visualized in LBs connected by radiating neurofilament+ elements, giving rise to a structure resembling a wheel (Fig. 4b) One ring localized around the central portion of the Ser129-p aSyn+ band, while a second ring surrounded the Ser129-p aSyn/beta-tubulin signals at their extreme periphery. The detailed distribution of cytoskeletal components around the Ser129-p aSyn+ band was best observed in 3D (Fig. 4d, Online Resource Video 6-S8).

The intricate organization of neurofilaments at the peripheral portion of LBs was commonly observed in onion skin-type morphologies in all PD patients analyzed in this study, suggesting that this is a characteristic feature of this LB-type (examples provided in Online Resource Fig. 5). For other cytoplasmic inclusions without ring-shaped appearance, the enrichment of immunolabeling for cytoskeletal markers was less prominent, although (diffuse) immunoreactivity was occasionally observed in these morphologies. In dystrophic LN morphologies such wheel-like arrangements of neurofilaments were not observed, although immunoreactive features could sometimes be observed at the extreme periphery of these structures, as previously described [32, 33]. Together, these findings suggest that the organization of Ser129-p aSyn and cytoskeletal markers in peripheral cage-like framework was characteristic for a specific subset of LBs, possibly indicating that the recruitment and structured alignment of cytoskeletal components at the periphery of LBs is particularly associated with certain maturation stages.

### Indications for enrichment of lipids and proteins in the core of LB-like inclusions

The presence of a structured-appearing peripheral framework enriched in cytoskeletal components and Ser129-p aSyn in the subset of onion skin-type nigral LBs points to the possibility that these components play a role in encapsulating accumulated material in the LB core. This inspired us to explore the distribution of proteins and lipids, the major components of LBs, within these structures. We recently demonstrated that proteins and lipids can be detected in aSyn+ LBs using a label-free nonlinear optical imaging technique, coherent anti-Stokes Raman scattering (CARS) in combination with confocal microscopy [67] and here we took further advantage of the resolution of this technique (~ 300 nm) which allows to separate between LB center versus periphery.

**Fig. 5:**
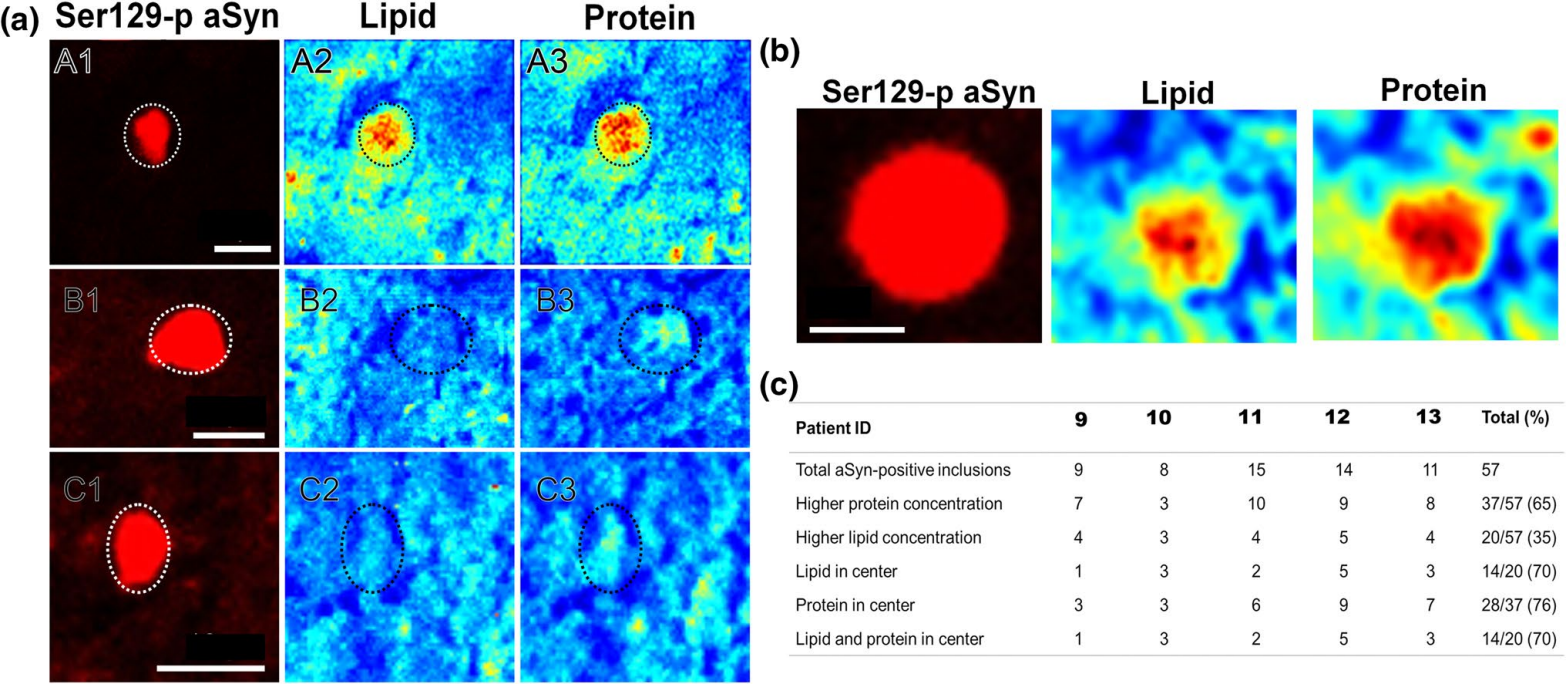
Protein and lipid distribution of nigral LBs by CARS microscopy. Applied Ser129-p aSyn antibody: Abcam ab59264. **a–c** Different LB compositions as identified by CARS microscopy. Ser129-p aSyn+ inclusions are depicted in the first column and CARS signal intensities at 2850 cm^−1^ and 2930 cm^−1^ shows their lipid (second column) and protein (third column) distributions, respectively. Low CARS intensities are depicted in blue, whereas high intensities are depicted in red. LBs with different compositions were identified: LBs with high CARS intensities for proteins and lipids compared to the direct environment (top row), with high CARS intensity for proteins but not lipids middle row), and with low CARS intensity for proteins and lipids (bottom row). **b** Representative image of a LB with high protein and lipid signal centralized in the structure. **c** Numbers and proportions of nigral LBs with high (centralized) lipids or proteins per patient. In total 57 LBs were observed in 5 PDD patients of which 37 showed high protein concentration and 20 showed high lipid concentration compared to surrounding tissue. In total 14 out of 20 with high lipid concentration showed lipids mainly in the center, whereas 28 out of 37 showed mainly proteins in the center. **a** Scale bar = 10 µm; **b**: Scale bar = 5 µm

We applied a similar pipeline (Online Resource Fig. 9) in SN tissue of 5 PD patients (specified in Online Resource Table 1). In short, dried sections of fresh-frozen midbrain tissue blocks including the SN were scanned by CARS microscopy, after which immunofluorescent labeling for Ser129-p aSyn was done on the same section to allocate somatic aSyn+ inclusions. We included a total of 57 inclusions with diameters larger than 5 µm in our analysis. Subclassification of LB morphologies was not feasible in the native specimens as their morphological integrity was impacted after scanning. Likely partially because of this reason, aSyn inclusions analyzed by CARS revealed substantial heterogeneity in their lipid/protein composition, both within and between patients (Fig. 5c). Among the scanned inclusions were structures displaying higher levels for both proteins and lipids compared to the direct surrounding, inclusions with higher protein levels without increased lipid levels, and labeled inclusions for which no differences were detected in protein and lipid levels compared to their surrounding (Fig. 5a). The majority of scanned LBs showed higher protein levels (37 out of 57) compared to their surrounding, while higher lipid content was detected in 20 out of 57 inclusions (Fig. 5c). When considering the subset of inclusions with increased protein compared to the direct surrounding, we observed that proteins were most frequently (28 out of 37 inclusions) clustered in the central portion of inclusions. A similar trend was observed for lipids, as lipid content was found enriched in the core in 14 out of 20 analyzed inclusions in which enriched lipids were detected (Fig. 5c). Of note, in all 14 inclusions with increased centralized lipids, their local enrichment was accompanied by increased protein content in the core (example in Fig. 5b). Together, our CARS results provide evidence for a central clustering of accumulated lipids and proteins in Ser129-p aSyn+ structures, supporting the hypothesis that such components are encapsulated in LBs.

### Ser129-p and CTT aSyn demonstrate distinct subcellular profiles

**Fig. 6:**
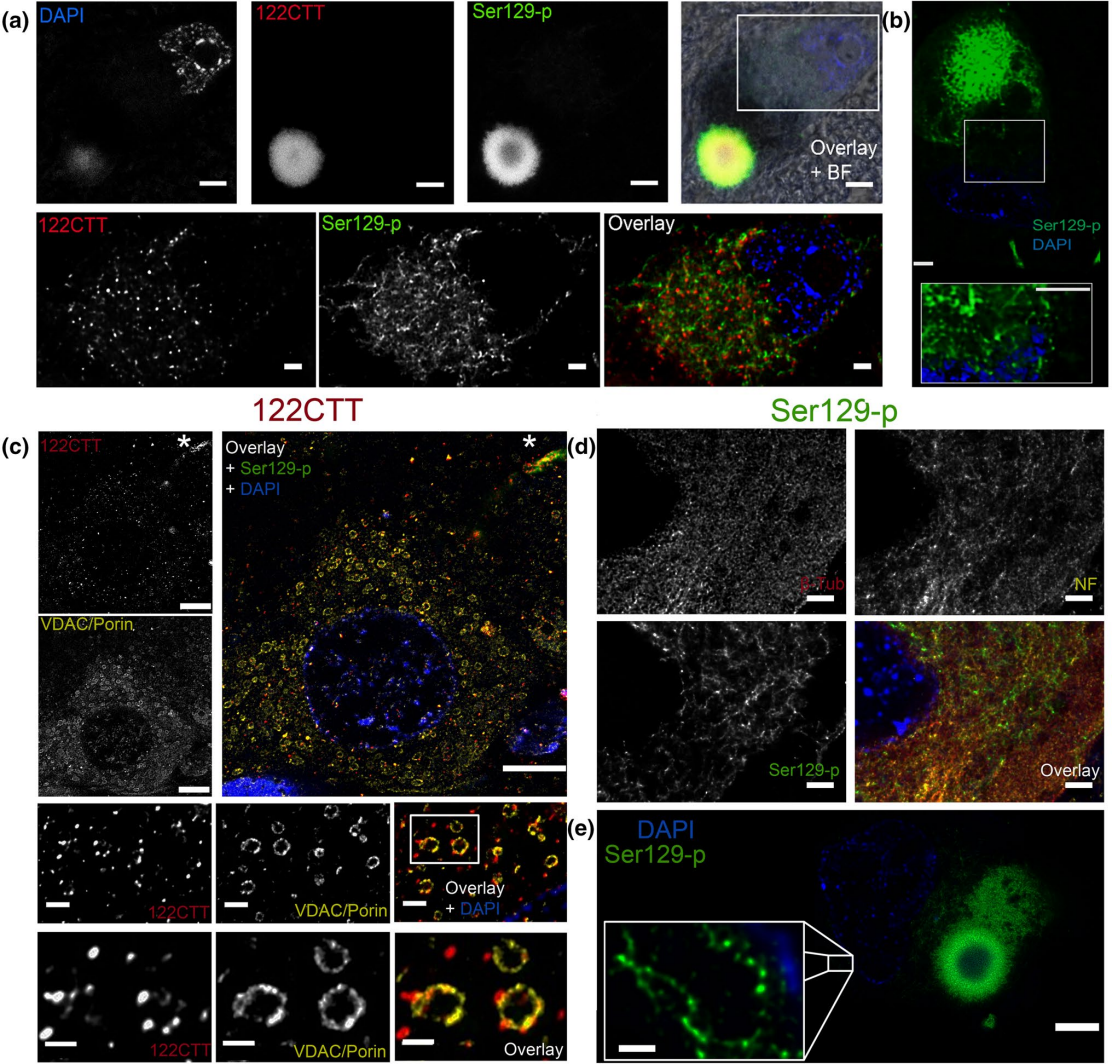
Subcellular manifestation of features containing 122CTT and Ser129-p aSyn immunoreactivity. Antibodies shown in all images: 122CTT: asyn-134; Ser129-p aSyn: asyn-142 **a** Overview of a neuromelanin-containing dopaminergic neuron in the SN with a LB (upper row) and a zoom-in on immunoreactive profiles in its cytoplasm (lower row). The signal intensity for 122CTT and Ser129-p aSyn was highest in the LB, suggesting an enrichment for these PTMs in pathological inclusions, while less intense cytoplasmic immunoreactivity could also be observed for 122CTT and Ser129-p aSyn showing different manifestations (lower panels). **b** example of a cortical neuron (TEC) demonstrating a uniformly labeled inclusion in combination with perinuclear Ser129-p aSyn+ network profiles. **c** Cytoplasmic 122CTT aSyn+ punctae showed association with VDAC/Porin-labeled mitochondria in the hippocampal CA2 of a PD patient. Labeling of a LN by CTT aSyn is indicated with an asterix. **d** Ser129-p aSyn+ network profiles showed only partial overlap with intracytoplasmic networks visualized by markers for beta-tubulin and neurofilament in the cytoplasm of a neuromelanin-containing dopaminergic neuron in the SN of a PD patient. **e** Localization of 122CTT aSyn+ punctae at the outer membrane of mitochondria in the hippocampal CA2 of a PD patient at different magnifications. **e** Dopaminergic neuron in the SN containing a combination of uniform inclusion and onion skin-type LB surrounded by a Ser129-p aSyn+ network. Inset: higher magnification of Ser129-p aSyn+ profiles with a diameter of ~ 70 nm. **a**, **b** Deconvolved CSLM images; **c-f:** deconvolved STED images. **a** Upper row: scale bar = 5 µm; lower row: scale bar = 2 µm; **b**: scale bar = 5 µm; **c**: scale bar = 2 µm; **d**: upper row: scale bar = 1 µm; lower row: scale bar = 0.5 µm; **e**: main Figure: scale bar = 5 µm; inset: scale bar = 0.5 µm

In addition to the expected enrichment of Ser129-p and CTT aSyn proteoforms in LBs based on previous results [3, 27, 62], using our sensitive imaging setup we also observed robust immunoreactive profiles in the neuronal cytoplasm distant from these inclusions for all aSyn antibodies, for which we hence characterized their distribution and localization with subcellular features using STED microscopy. As expected based on the well-reported localization of aSyn at presynaptic terminals [7, 25, 31, 49] and previously discussed brightfield microscopy observations (Online Resource Fig. 1), antibodies raised against aSyn domains revealed immunoreactivity in the neuropil with considerably less labeling of the cell body in PD patients and controls consistent with synaptic-like staining patterns (Online Resource Fig. 7a) [39]. In contrast, such synaptic profiles were less pronounced in labelings for Ser129-p and CTT aSyn (Online Resource Fig. 7a), for which immunoreactivity was found to be localized mainly in neuronal cell bodies (Fig. 6a, Online Resource Fig. 7b). For 119CTT, limited immunoreactivity outside Lewy bodies was observed even at high antibody concentrations, suggesting that this aSyn fragment may be particularly associated with or enriched in pathological inclusions.

The cytoplasmic subcellular immunoreactivity patterns for Ser129-p and 122CTT aSyn showed limited colocalization and differed in their appearance (Fig. 6a). In particular, 122CTT aSyn revealed small immunoreactive punctae throughout the neuronal cytoplasm, while Ser129-p aSyn immunoreactivity visualized a cytoplasmic network (Fig. 6a). This network was strongest in the perinuclear area—surrounding a nucleus devoid of labeling—and sometimes in continuation with similar Ser129-p aSyn+ features in the proximal portion of neuronal processes.

#### 122CTT aSyn+ punctae are associated with mitochondria

122CTT aSyn+ punctae were not only observed in PD patients, but also in control donors without Lewy pathology (Online Resource Fig. 7a). These punctate profiles appeared more pronounced in the hippocampus and transentorhinal cortex compared to the SN (Fig. 6c). We observed cytoplasmic reactivity patterns of 122CTT aSyn using different antibodies against this epitope (syn105 and asyn-134). Co-visualization of 122CTT aSyn with subcellular markers revealed that 122CTT aSyn+ punctae in the cytoplasm of neurons followed the distribution of mitochondria, and co-localized with mitochondrial morphologies immunoreactive for Porin/VDAC, a marker for the outer mitochondrial membrane (Fig. 6c). The localization of 122CTT aSyn+ punctae at mitochondrial membranes was confirmed in 3D reconstructions of STED images (Online Resource Video 10). Together, our findings suggest 122CTT aSyn is generated in donors with and without PD, which is in line with earlier discussed WB results (Online Resource Fig. 1c, d) and previous literature suggesting the presence of CTT aSyn under physiological conditions [44, 52].

**Fig. 7:**
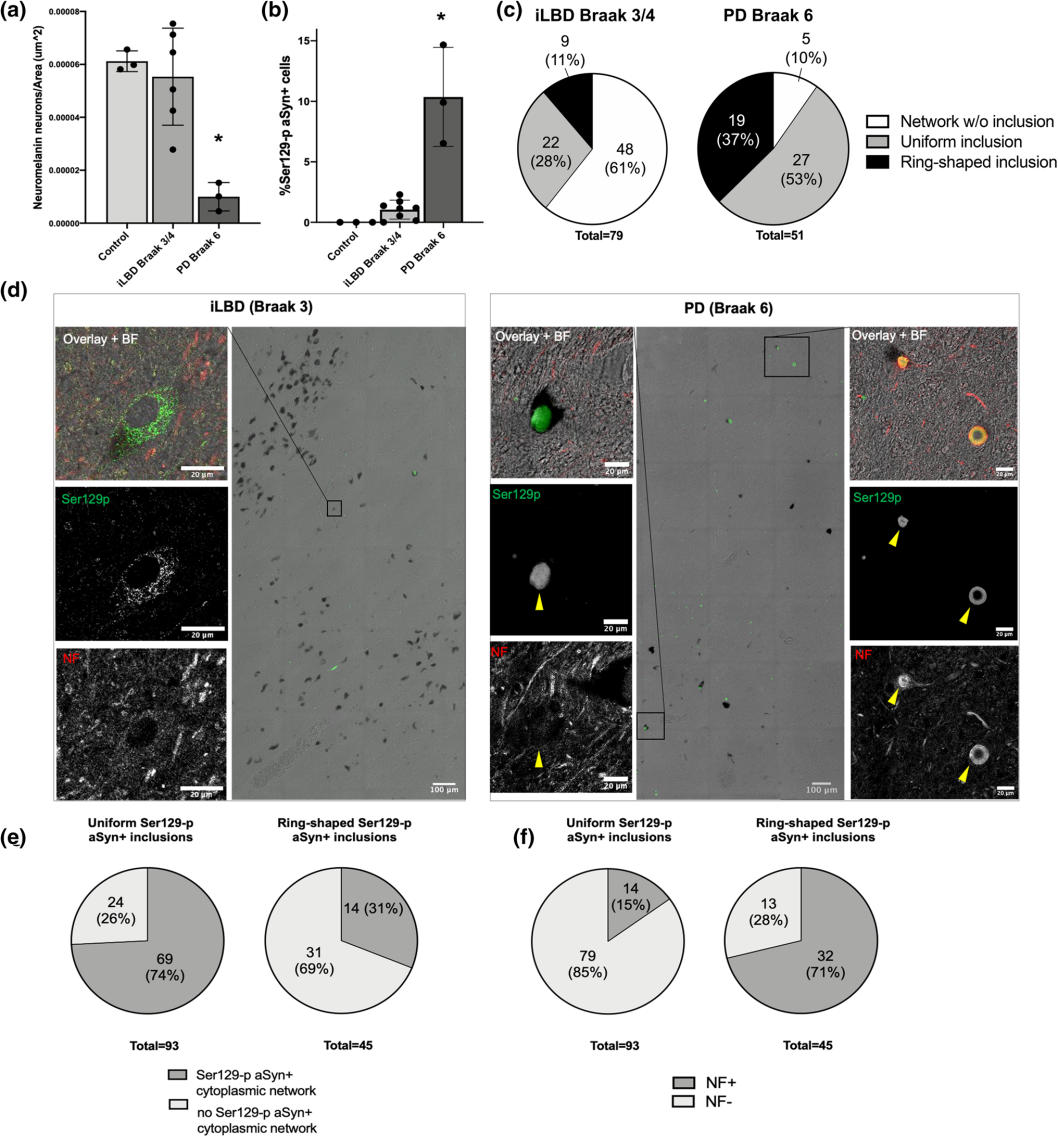
Ser129-p aSyn+ cytoplasmic network profiles are associated with early stages of aSyn pathology. aSyn antibody shown: Ser129-p aSyn: asyn-142. **a, b** Quantification of the number of neuromelanin-containing neurons (**a**) and proportion of Ser129-p aSyn+ neurons (**b**) in midbrain sections of controls, iLBD donors and PD patients. **c** frequency distribution of selected neuronal Ser129-p aSyn+ profiles in iLBD donors and PD patients. **d** Representative overview images in the SN of an iLBD Braak stage 3 donor and a PD Braak stage 6 donor. Insets display examples of Ser129-p aSyn+ cytoplasmic network profiles in a neuron without apparent inclusion in the iLBD donor, and LBs with uniform (left) and ring-shaped (right) appearances in the PD patient. In addition, associated neurofilament distributions are shown. **e** Frequency of Ser129-p aSyn+ cytoplasmic network profiles in neurons containing either uniform or ring-shaped Ser129-p aSyn + inclusions. **f** Frequency of neurofilament immunoreactivity in uniform or ring-shaped Ser129-p aSyn+ inclusions

#### Ser129-p aSyn is incorporated in a disease-associated cytoplasmic network

**Fig. 8:**
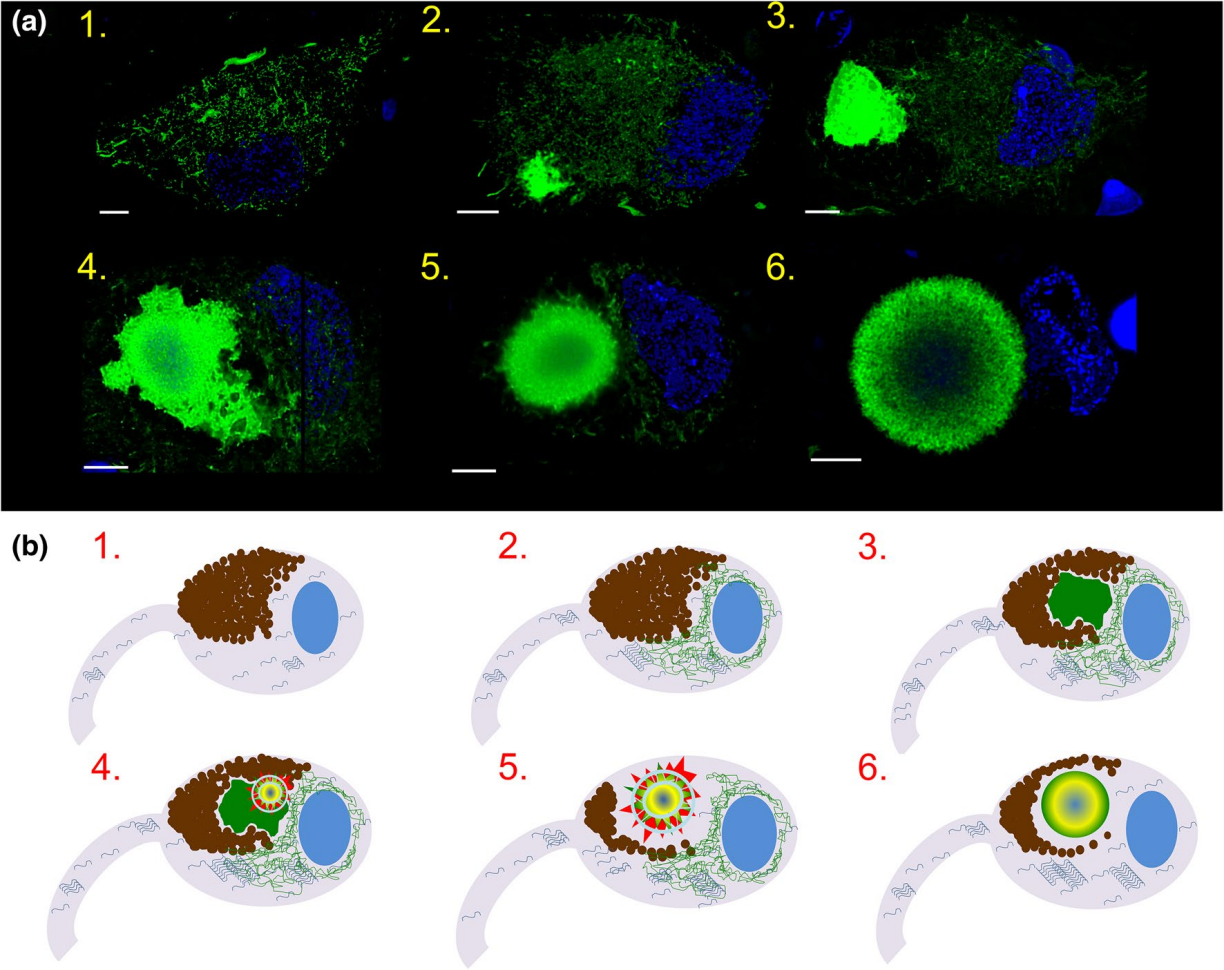
Various intracytoplasmic appearances of Ser129-p aSyn hint towards different maturation stages of LBs. **a** Different patterns of Ser129-p aSyn immunoreactivity in neuromelanin-containing dopaminergic cells in the SN of PD patients. Images are 2D Maximum projections of 3D reconstructions based on image stacks, made in adjacent tissue sections. Ser129-p aSyn antibody: asyn-142. The following cellular phenotypes were commonly observed: 1) neurons containing cytoplasmic Ser129-p aSyn+ network profiles without apparent inclusion; 2,3) neurons with cytoplasmic Ser129-p aSyn+ network profiles and smaller (2) and larger (3) uniformly labeled inclusions; 4) neuron with cytoplasmic Ser129-p aSyn+ network profiles and a combination of uniformly labeled inclusion and onion skin-type LBs; 5) neuron with cytoplasmic Ser129-p aSyn+ network profiles and ring-shaped LB; 6) neuron containing a ring-shaped LB with limited cytoplasmic Ser129-p aSyn+ network profiles. Scale bars: 5 µm. **b** Hypothetical sequence of events in LB formation in dopaminergic neurons in the SN of PD patients, based on Ser129-p aSyn immunoreactivity patterns (visualized in green; detailed explanation in text). 1) Neuromelanin-containing neuron under conditions of aSyn homeostasis; 2) alignment of Ser129-p aSyn into a cytoplasmic network; 3) sequestration of proteins into expanding uniform Ser129-p aSyn+ inclusion; 4,5) recruitment of cytoskeletal structures to restructure the inclusion in a compaction-like manner, resulting in a multilamellar onion skin-type LB morphology (6) reflecting the encapsulation of highly aggregated proteins and lipids in its core

The network-like Ser129-p aSyn immunoreactivity was best visible in cell bodies of large neuromelanin-containing neurons in the SN (Fig. 6d), but also in other cell types, such as pyramidal neurons in the hippocampus and transentorhinal cortex (Fig. 6b, Online Resource Fig. 8c). Most often, cytoplasmic Ser129-p aSyn+ network profiles were observed in neurons containing a smaller or expansive-appearing inclusion uniformly stained for Ser129-p aSyn, and in a smaller fraction of neurons containing onion skin-type LBs. However, features could also be observed in certain neurons without apparent inclusion in PD patients (e.g. Fig. 8a, 1). Many other neurons without inclusion in donors with PD did not reveal cytoplasmic Ser129-p aSyn+ network profiles, while no Ser129 aSyn+ features were observed in donors without Lewy pathology (Online Resource Fig. 7b)—which is consistent with the observation that Ser129-p aSyn was not detected in control tissue by WB (Online Resource Fig. 1c). Therefore, we conclude that the alignment of Ser129-p aSyn in a cytoplasmic network is associated with pathological conditions.

Although some overlap was observed between the Ser129-p aSyn network with cytoplasmic distributions of intermediate neurofilaments and beta-tubulin (Fig. 6d)—and to even lesser extent calreticulin, a marker for the endoplasmic reticulum (Online Resource Fig. 6)—localization analyses by STED demonstrated that the cytoplasmic alignment of Ser129-p positive elements could not be fully explained by its colocalization to these intracellular networks. However, this does not exclude the possibility that Ser129-p aSyn localizes to parts of these and also other cytoplasmic networks. The observed diameter of the aligned structures in the network was generally ~ 70 nm under the applied STED scan settings.

### Ser129-p aSyn+ cytoplasmic network profiles are associated with early disease stages

Importantly, cytoplasmic Ser129-p aSyn+ profiles were not only observed in end-stage PD patients, but also in the SN of patients with early PD/iLBD donors (Braak 3/4, Online Resource Fig. 8a, b) and in the middle temporal gyrus of Braak 5-staged PD/iLBD donors (Online Resource Fig. 8c) [6]. This points to the possibility that the Ser129-p aSyn+ network manifests already at early stages of LB formation and maturation. We explored this hypothesis by quantifying different neuronal Ser129-p aSyn+ profiles in neurons in high-resolution CSLM tile scans of the SNpc in midbrain sections of 3 donors with advanced PD (Braak 6), 6 donors with iLBD (Braak 3/4) as well as 3 non-neurological controls. iLBD donors have a relative sparing of dopaminergic neurons compared to advanced PD patients in combination with limited local aSyn pathology, which is therefore considered to represent early pathological changes. We classified different Ser129-p aSyn+ profiles in individual neuromelanin-containing neurons, for which we distinguished between neurons with Ser129-p aSyn+ cytoplasmic network profiles in absence of LB-like inclusions, neurons bearing uniform Ser129-p aSyn+ inclusions with or without cytoplasmic network profiles, and neurons bearing ring-shaped (onion skin-type) Ser129-p aSyn+ inclusions with or without cytoplasmic network profiles. The relative abundance of these immunoreactivity patterns among the total pool of Ser129-p aSyn + neurons was compared between groups of iLBD and PD donors.

As expected, more neuromelanin-containing dopaminergic neurons were observed in iLBD donors compared to PD patients (Fig. 7a, *t* = 4.1, *p* = 0.005) in the analyzed brain sections- reflecting more severe neuronal loss in PD patients—while the proportion of identified Ser129-p aSyn+ neurons was significantly lower (Fig. 7b, *t* = 6.7, *p* < 0.001) in iLBD donors compared to the PD patients in our analysis. Interestingly, the frequency distributions of the selected neuronal Ser129-p aSyn+ profiles showed marked differences between donors with iLBD versus PD (Fig. 7c), as the proportion of neurons with a cytoplasmic Ser129p aSyn+ network without apparent inclusion was substantially higher in iLBD (61%) than in advanced PD (10%). This finding supports that cytoplasmic Ser129-p aSyn + network profiles in neurons without inclusions are associated more with early than with late pathological Braak stages. In contrast, the observed proportion of ring-shaped Ser129-p aSyn + inclusions was higher in PD than in iLBD, suggesting this LB subtype may be particularly associated with later disease stages (Fig. 7c). Although less frequent, however, ring-shaped appearances were also observed in iLBD donors.

### Inclusion subtypes display differential association with cytoplasmic Ser129 + aSyn network profiles and neurofilament distributions

For all uniformly labeled (*N* = 93) and ring-shaped (*N* = 53) Ser129-p aSyn+ inclusions > 5 µm identified in iLBD donors and PD patients in the analysis described above, we studied their association with a cytoplasmic Ser129-p aSyn+ network in the same neuron and the presence of neurofilament+ profiles in the inclusion. We found that the great majority of ring-shaped inclusions (71%) in our analysis contained neurofilament+ (e.g. cage-like) profiles while neurofilament enrichment was observed only in a smaller subset of the analyzed uniform inclusions (15%, Fig. 7d, f). Vice versa, cytoplasmic Ser129-p aSyn+ network profiles were more frequently observed in neurons with uniform inclusions (74%) than in neurons with ring-shaped inclusion types (31%, Fig. 7d, e). These data provide quantitative support for some of our observations in higher-resolution STED experiments that were mentioned earlier in this study. As we found indications that cytoplasmic Ser129-p aSyn+ network profiles are commonly observed at early disease stage, a differential association between uniform and ring-shaped inclusions with such profiles points to the possibility that they represent earlier and later LB maturation stages, respectively. Following this line of thought, the incorporation and organization of neurofilaments in a cage-like framework as observed in ring-like structures may be mainly associated with mature inclusion types.

### Pathology-associated phenotypes in neuromelanin-containing neurons based on subcellular Ser129-p aSyn + features

By comparing Ser129-p aSyn+ features in neurons using different antibodies within and between patients, we identified certain commonly observed subcellular profiles of immunoreactivity in neuromelanin-containing neurons of brains with Lewy pathology. These subcellular phenotypes were all strongly associated with (early) pathology, as they were observed in patients with end-stage PD and in iLBD but not in any of the analyzed neurons in non-neurological control subjects. Representative examples are summarized in Fig. 8a, as visualized by maximum projections of CSLM 3D reconstructions of neuromelanin-containing nigral neurons in adjacent brain sections of the same PD patient. Commonly observed cellular phenotypes in the SN of PD patients based on Ser129-p aSyn immunoreactivity included: 1) neurons with Ser129-p aSyn+ cytoplasmic network but without apparent focal inclusion; 2/3) neurons displaying this network and smaller compact (2) or larger expansive-appearing (3) inclusions uniformly labeled for Ser129-p aSyn; 4) neurons revealing a cytoplasmic Ser129-p aSyn+ network and (a combination of) uniformly stained and onion skin-like inclusions; 5) neurons with a cytoplasmic Ser129-p aSyn+ network and ring-shaped inclusions; 6) neurons with a mature ring-shaped inclusion, but limited cytoplasmic Ser129-p aSyn+ network profiles.

## Discussion

A better understanding of the organizational structure of LBs in the human brain, the pathological hallmark of devastating neurodegenerative diseases such as PD and DLB, can yield important insights into the cellular processes involved in their formation and provide a reference point for translation of findings in novel cellular or animal models of PD to human disease. Microscopically, LBs are characterized by the enriched presence of aSyn, predominantly in the form of extensively PTM protein variants such as CTT and Ser129-p aSyn species [3, 21, 62]. In a recently published study by our group [67], we used correlative light and electron microscopy to identify ultrastructural components of aSyn-immunolabeled LBs in (ultra)thin brain sections, and demonstrated that they are abundant in cellular organelles and membranes. However, the detailed distribution of specific variants of aSyn, one of their major protein components, in this crowded LB landscape is not clear. In this study, we aimed to map the subcellular distribution of different aSyn proteoforms within the 3D architecture of LBs and their cytoplasmic localization distant from these structures. For this purpose, we developed and characterized antibodies against specific proteoforms and domains of the aSyn protein and applied them in immunolabelings on thicker (e.g. 20 µm) human brain-tissue sections in combination with high- and super-resolution light microscopy.

Although many neuronal pathological aSyn+ inclusions appeared heterogeneous and relatively unstructured as they revealed diffuse or uniform labeling patterns for different aSyn antibodies and other markers, a subset of nigral LBs demonstrated a remarkably consistent 3D onion skin-like organization consisting of concentric lamellar bands enriched for specific aSyn epitopes. This pattern was observed and quantified in multiple LBs of different PD patients. A multilamellar appearance of LBs has been described in previous studies that focused on LB ultrastructure using EM techniques [14, 20], while lamination patterns in LBs and dystrophic LNs were also suggested in studies using brightfield and fluorescent light microscopy approaches [22, 32, 33, 62]. Interestingly, in the present study this lamellar phenotype was visualized by a gradual distribution of immunoreactivities for antibodies directed against different proteoforms and also different domains of a single protein, aSyn. In such onion skin-type LBs, a different localization was consistently observed for 122CTT and Ser129-p aSyn proteoforms using the antibodies Syn105 and 11A5, thereby replicating previous findings by Prasad et al. [62]. In addition, we confirmed the differential localization of Ser129-p and CTT aSyn in onion skin-type LBs using new highly-selective monoclonal antibodies against the same epitopes and an antibody directed against another CTT variant (119CTT aSyn).

At the periphery of onion skin-type LBs, Ser129-p aSyn immunoreactivity was associated with a framework of cytoskeletal components such as beta-tubulin and intermediate neurofilaments, which are major constituents in organizing cellular organelles and substructures. The enrichment of cytoskeletal elements in LBs has historically been reported and neurofilaments were long thought to be a major component of LBs (reviewed in [41]), while their predominant immunoreactivity at periphery of LBs and LNs was already suggested in previous studies [32, 33]. Our higher-resolution imaging approach add to these findings the recurring intricate organization of neurofilaments in a subset of mature-appearing LBs and demonstrate their close association to Ser129-p aSyn and other cytoskeletal elements such as beta-tubulin. The structured arrangement of cytoskeletal elements at the peripheral portion of LBs indicates that these components are not just trapped in these inclusions, but may enable their organization and stabilization. A displacement of intermediate neurofilaments from their normal cellular distribution and their encapsulation of aggregated proteins have been previously described as consistent features of intracellular aggresome formation [29, 38]. Thereby, our results confirm previous studies proposing that LBs share phenotypic features with aggresomes [55], and indicate that the interplay of Ser129-p aSyn with cytoskeletal proteins is an important event in an extensive cellular regulation underlying LB maturation and morphogenesis in the PD human brain. A recent study in primary hippocampal neurons described that the formation of artificial intracellular inclusions triggered by administration of pre-formed fibrils (PFFs) of recombinant aSyn and visualized by microscopical techniques was associated with the biochemical enrichment of cytoskeletal components in insoluble fractions by proteomic approaches [48]. While it remains unclear to what extent these findings in experimental PFF models replicate the mechanisms leading to the formation and morphogenesis of bona fide Lewy pathology in humans, this finding suggests that the recruitment and organization of cytoskeletal components also occurs during the formation of proteinaceous accumulations of aSyn in vitro. Although not (directly) related to synucleinopathy, one study that combined pulldown assays with mass spectrometry in isolated synaptosomes from murine and non-diseased human brain reported that significantly more cytoskeletal elements were pulled down by Ser129-p aSyn peptides compared by unmodified aSyn peptides, suggesting that Ser129-p can change aSyn’s interactome leading to preferential interaction of aSyn with cytoskeletal components [50]. These findings, together with our observations in the PD brain, indicate that the interplay of Ser129-p aSyn proteoform with cytoskeletal elements could play an important role in LB maturation and stabilization.

Our description of a peripheral cytoskeletal framework of onion skin-type LBs—which was observed more frequently in late-stage PD than in iLBD—pointed to the possibility that their morphology reflects the encapsulation of accumulated cellular material. LBs have been reported to contain a plethora of protein components as well as lipids, many of which are likely derived from membranous organelles such as mitochondria, lysosomes/autophagosomes that are abundant in LBs [20, 67, 70]. Therefore, we explored protein and lipid content in nigral aSyn-labeled inclusion bodies in native sections by CARS microscopy- a label-free spectral imaging technique- in native tissue sections. Although the use of these morphologically less-preserved brain specimens did not allow detailed subclassification of specific LB morphologies, we still found recurring trends for centralization of proteins and lipids in the core of aSyn-labeled inclusion bodies in the SN. Our previous detection of increased lipid content of LBs by CARS was reproduced [67], while its association with the LB core support results from previous studies using different lipophilic dyes [12, 22, 30]. Moreover, one other study previously reported a similar result as it localized lipids to the core of the majority (58%) 12 nigral LBs that were identified by immunohistochemical detection of Ser129-p aSyn using subsequent Fourier transform infrared micro-spectroscopy (FTIR) in FFPE tissue sections from 3 PD patients [4]. Further development and validation of approaches to localize (at high resolution), quantify and classify lipid and protein content in post-mortem human brain-tissue specimens from donors with PD is important as the contribution of impaired lipid homeostasis to PD pathology – possibly resulting from aberrant aSyn-membrane interactions—is increasingly recognized [19, 67]. A recent study applied FTIR to Amyloid-β plaques in Alzheimer's disease and thereby reconstructed their respective development sequence [64], underlining the potential of the application of label-free imaging methods in human brain samples for a better understanding of the pathogenesis of neurodegenerative disease.

Outside of LBs, Ser129-p-aSyn immunoreactivity in a cytoplasmic network surrounding a nucleus without immunoreactivity was characteristic for PD and iLBD patients, but not observed in controls. Diffuse or granular cytoplasmic immunoreactivity has been described as a specific feature of certain antibodies against aSyn in different studies using light microscopy [2, 40] including antibodies with a proposed preferential reactivity for disease-associated aSyn [39, 63], and such features have been suggested to be associated with early stages of LB formation [70]. In support of this, we observed Ser129-p aSyn+ network profiles in neurons without apparent inclusions more frequently in iLBD Braak 3/4 donors than in advanced PD (Braak 6) patients. This finding suggest that the alignment of Ser129-p aSyn in a cytoplasmic network represent an early pathological phenotype possibly preceding LB formation. Interesting in this perspective are the results of a previous study, in which increased Ser129-p aSyn levels were measured by WB in soluble fractions of cingulate and temporal cortices prior to the histological detection of Lewy inclusions [46]. Involvement of Ser129-p aSyn early in inclusion formation is further supported by the findings that administration of recombinant (full-length and CTT) aSyn (PFFs) in cell culture leads to a rapid recruitment of soluble endogenous aSyn and its intracellular phosphorylation at Ser129 [47, 69]. A possible role of Ser129-p aSyn at this stage could be a stabilizing effect on accumulating proteins—e.g. Ser129-p was demonstrated to inhibit aSyn fibrillogenesis in vitro [56, 59]—while Ser129-p aSyn has also been suggested to serve as an activator of autophagic instruments [10, 58]. Together, these results are inconsistent with theories that Ser129-p aSyn only occurs after LB formation [56].

Based on different subcellular Ser129-p aSyn immunoreactivity patterns within and between PD patients with different Braak stages in our study, we identified a subset of commonly observed pathological phenotypes in nigral neuromelanin-containing neurons (Fig. 8a). As discussed earlier, we found indications that cytoplasmic Ser129-p aSyn+ network profiles are particularly associated with early disease stages, and such profiles were more frequently observed in cells with uniform Ser129-p aSyn inclusion types than in cells with ring-shaped LBs. This indicates that uniform inclusions may be more associated with early cytopathology. Ring-shaped (onion skin-type in 3D) LBs, in turn, may represent a later maturation stage of LBs, and commonly showed enrichment of neurofilaments in a cage-like framework.

In an attempt to integrate our high-resolution observations in post-mortem human brain tissue in a hypothetical sequence of events in LB maturation (Fig. 8b), we speculate that heterogeneous and unstructured early aSyn inclusions are shaped into consistent, more organized morphologies during their maturation in an attempt to the encapsulate accumulated proteins and lipids (Fig. 8b). In healthy neurons, basal proteolytic activity of intracellular protein degradation systems—in particular the ubiquitin-proteasomal system and (chaperone-mediated) autophagy—maintains protein and lipid homeostasis. If this balance is disrupted, this could result in the accumulation of aSyn, other proteins, membranes and/or organelles within neurons [67]. Phosphorylation of aSyn may take place at this stage to i.e. to activate the macroautophagic machinery [58] and/or stabilize intracellular accumulations of damaged proteins and organelles. The convergence of Ser129-p aSyn in a cytoplasmic network surrounding the nucleus (step 2) may reflect the sequestration of aggregated material to the perinuclear area for the formation of aggresomes. When the growing aggresome cannot be degraded by aggrephagy over time (step 3), cytoskeletal systems may be recruited to the LB to actively restructure the inclusion into a compact and stable morphology (step 4, 5). This idea of restructuring of LBs during their maturation in a compaction-like manner has been proposed before in literature [40]. In the mature onion skin-like LB morphology, highly aggregated proteins and lipids—largely derived from cellular material such as organelles [67]—are centralized in the core of the structure, encapsulated by a cage-like framework of Ser129-p aSyn and cytoskeletal components (step 5, 6). This hypothetical sequence of events in LB maturation needs to be explored in future experimental studies, for instance in cellular or animal models of LB formation [48]. Similarly, since our observation and hypothetical sequence is mainly based on nigral LBs in PD, it also needs to be analyzed if a similar process could be applicable to LBs from other brain regions or in other synucleinopathies, including DLB. In support of a similar process in different brain regions we commonly observed cytoplasmic Ser129-p aSyn+ network profiles in cortical neurons, for example in an iLBD donor and PD patients with Braak 5 (Online Resource Fig. 8c). Based on the results of our explorative comparisons of LB subtypes in iLBD donors and PD patients, we hypothesize that cytoskeletal elements—including neurofilaments—may play a regulatory role in the morphogenesis of onion skin-type LBs at later maturation stages. The incorporation and organization of neurofilaments in a subset of mature LBs may also explain our previous observation that radiating filamentous structures are present in a smaller subset of aSyn+ LBs by CLEM [67].

In line with previous studies using antibodies against CTT aSyn species, we found two CTT proteoforms to localize towards the core of LBs [15, 52, 62], while CTT of aSyn was repeatedly found to increase the propensity of aSyn to form amyloid aggregates in vitro [44, 45, 53, 71]. These observations could be perceived as support for a role of CTT aSyn driving aSyn aggregation and leading to proteinaceous LBs, as was proposed in [62]. However, as LBs contain a medley of fragmented organelles [67], including components that are able to cleave aSyn—for instance lysosomal hydrolases such as cathepsins [9], caspase-1 [71], or calpains [15]- it cannot be ruled out that CTT Syn is generated in the core of LBs and LNs at later maturation stages. Our WB results on human brain-tissue specimens could be placed in line with this last notion, as they demonstrated the incorporation of CTT fragments mainly in highly aggregated, HMW HEPES/sucrose-insoluble complexes associated with DLB pathology, in contrast to Ser129-p aSyn which showed a wider spectrum of insoluble species in DLB patients (Online Resource Fig. 1). Accordingly, antibodies against Ser129-p aSyn revealed the largest variety of pathology-associated morphologies in the PD brain. Interestingly, the intracellular CTT of aSyn PFFs after their administration to neurons (primarily at Glu-114) was reported to represent an important step in their cellular processing and efficient packing in inclusion bodies [47]. These results are in further support of a proposed extensive cellular regulation of Lewy inclusion formation and maturation, and indicate an important role for aSyn PTMs in this process.

We describe punctate reactivity for different antibodies against 122CTT aSyn in the neuronal cytoplasm, which was independent of the presence of Lewy pathology in PD patients and also observed in controls. This suggests that the generation of this PTM could be a normal cellular process, which is in line with WB detection of CTT aSyn in non-diseased brains in our study and previous studies [44, 52]. Unlike antibodies against NT, NAC and CT aSyn, 122CTT aSyn+ punctae were prominently present in the cell body and less associated with synaptic-like staining patterns. Of note here is that an important role was recently attributed to the CT of aSyn—in particular its interaction with calcium- in the localization of aSyn at presynaptic terminals [43], possibly explaining different localization patterns of CTT aSyn compared to proteoforms with an intact CT. Part of the 122CTT aSyn+ punctae in the cytoplasm localized at the outer membrane of VDAC/Porin-reactive mitochondria. This could be placed in line with the finding that CTT aSyn was detected mainly in fractions enriched for lysosomes and mitochondria derived from SH-SY5Y cells overexpressing human WT α-synuclein [52]. Future experimental studies are necessary to explore the functional relevance of the localization of 122CTT aSyn to mitochondria.

An intriguing and consistent observation was that the core of onion skin-type LBs showed converging immunoreactivity for DAPI—a dye that binds to T-A-rich regions of DNA [72], although this signal in LBs was weaker than in nuclei. This characteristic of LBs has already been reported before [61]. Although at this point the relevance of this observation is not clear, it was speculated that this may be the result of mitochondrial DNA incorporated in LBs [61]. The idea that damaged DNA is integrated in LBs is conceivable, given a recently described role of aSyn in DNA repair processes [65]. Alternatively, however, DAPI reactivity in LBs may be explained by nonselective interaction of DAPI with certain aggregated proteins or lipids in the center of LBs.

In summary, the present study provides a STED perspective on the subcellular arrangement of Ser129-p and CTT aSyn proteoforms and the architecture of Lewy pathology. Our results reveal a consistent onion skin-like distribution of different aSyn proteoforms in a subset of nigral LBs. The structured-appearing organization of Ser129-p aSyn with cytoskeletal components at the periphery of such onion skin-type LBs suggests that their morphology reflects the encapsulation of accumulated proteins and lipids. Analysis of detailed immunoreactivity patterns of aSyn-specific antibodies led us to the identification of different pathology-associated, subcellular phenotypes of aSyn distribution and accumulation. Based on our findings, we propose a hypothetical sequence of events in Lewy inclusion formation and maturation in which Ser129-p aSyn may have a central role, which needs to be tested in well-characterized experimental models. The applied combination of selective antibodies and advanced microscopy techniques in this study allowed for a phenotyping of antibody-labeled aSyn cytopathology in great detail, which opens exciting opportunities for better characterization and understanding of LB formation in the pathogenesis of PD.

## Supplementary Information

Below is the link to the electronic supplementary material.

Supplementary file1 (MP4 27030 kb)

Supplementary file2 (MP4 6268 kb)

Supplementary file3 (MP4 51129 kb)

Supplementary file4 (MP4 50860 kb)

Supplementary file5 (MP5 28269 kb)

Supplementary file6 (MP4 4794 kb)

Supplementary file7 (MP4 6312 kb)

Supplementary file8 (MP 448653 kb)

Supplementary file9 (MP4 21411 kb)

Supplementary file10 (MP4 24531 kb)

Supplementary file11 (PDF 11197 kb)
