# Supplementary material for: The subcellular arrangement of alpha-synuclein proteoforms in the Parkinson’s disease brain as revealed by multicolor STED microscopy
Source: Acta Neuropathol. 2021 Jun 11;142(3):423–48. doi: 10.1007/s00401-021-02329-9 (PMC8357756; doi:10.1007/s00401-021-02329-9)
Supplement: Supplementary file 11 — Supplementary file11 (PDF 11197 kb) [file 401_2021_2329_MOESM11_ESM.pdf]

Online Resource Material

The subcellular arrangement of alpha-synuclein proteoforms in the Parkinson's disease brain as revealed by multicolor STED microscopy

Tim E. Moors[1], Christina A. Maat[1], Daniel Niedieker[2], Daniel Mona[3], Dennis Petersen[2], Evelien Timmermans-Huisman [1], Jeroen Kole[4], Samir F. El-Mashtoly[2], Liz Spycher[3], Wagner Zago[5], Robin Barbour[5], Olaf Mundigl[6], Klaus Kaluza[6], Sylwia Huber[7], Melanie N. Hug[7], Thomas Kremer[3], Mirko Ritter[8], Sebastian Dziadek[9], Jeroen J.G. Geurts[1], Klaus Gerwert[2], Markus Britschgi[3], Wilma D.J. van de Berg[1*]*

[1]Amsterdam UMC, location VU University Medical Center, Department of Anatomy and Neurosciences, Section Clinical Neuroanatomy, Amsterdam Neuroscience, De Boelelaan 1108, 1081 HZ Amsterdam, The Netherlands

[2] Department of Biophysics, Ruhr-University Bochum, Bochum, Germany, Universitätsstrasse 150, 44801 Bochum

[3] Roche Pharma Research and Early Development; Neuroscience and Rare Diseases Discovery and Translational Area, Roche Innovation Center Basel, Grenzacherstrasse 124, CH – 4070, Basel, Switzerland

[4] Amsterdam UMC, location VU University Medical Center, Department of Physiology, De Boelelaan 1108, 1081 HZ Amsterdam, The Netherlands.

[5] Prothena Biosciences Inc, South San Francisco, California, CA, 94080, USA

[6] Roche Pharma Research and Early Development; Therapeutic Modalities; Large Molecule Research, Roche Innovation Center Munich, Nonnenwald 2, D-82377 Penzberg, Germany

[7] Roche Pharma Research and Early Development; Therapeutic Modalities; Small Molecule Research, Roche Innovation Center Basel, Grenzacherstrasse 124, CH – 4070, Basel, Switzerland

[8] Roche Diagnostics GmbH, Nonnenwald 2, D-82377 Penzberg, Germany

[9] Roche Pharma Research and Early Development; Oncology Discovery and Translational Area, Roche Innovation Center Basel, Grenzacherstrasse 124, CH – 4070, Basel, Switzerland

[10] Ann Romney Center for Neurologic Diseases, Department of Neurology, Brigham and Women's Hospital and Harvard Medical School, Boston, MA 02115, USA

***Corresponding authors:**

Wilma D.J. van de Berg, PhD

Dept. of Anatomy & Neurosciences, chair section Clinical Neuroanatomy and Biobanking

Amsterdam Neuroscience, Amsterdam UMC, location VU University Medical Center

O2 building, room 13 E11, De Boelelaan 1108, 1081 HZ Amsterdam, the Netherlands.

e-mail: wdj.vandeberg@amsterdamumc.nl; Tel.nr: +316-25694907

Markus Britschgi, PhD

Roche Pharma Research and Early Development; Neuroscience and Rare Diseases Discovery and Translational Area, Roche Innovation Center Basel, Grenzacherstrasse 124, CH – 4070, Basel, Switzerland.

e-mail: markus.britschgi@roche.com; Tel. nr: +41 61 687 91 16

Online Resource Methods

Generation and detailed description of characterization of aSyn specific antibodies

Generation, screening and initial characterization

Generation and characterization of the antibodies 11A5 and syn105 was previously described [1, 2, 3]. Additional novel antibodies were generated by immunizing rabbits either with E. coli derived recombinant full length aggregated aSyn (asyn-055 and asyn-058) or KLH-conjugated peptides representing the C-terminus of 119CTT, 122CTT aSyn, or aSyn derived peptide phosphorylated at Ser129, respectively (for asyn-131, asyn-134, asyn-142; Online Resource Fig. 1, Online Resource Table 5). After screening of serum titers, standard B cell cloning was performed to generate rabbit monoclonal antibodies (mAbs). Recombinant mAbs were screened for binding to the peptides representing the aa1-60, 61-95, and aa96-140 by ELISA, respectively (for asyn-055 and asyn-058), or the C-terminus of aSyn119CTT, aSyn122CTT or phosphorylated at Ser129, respectively (for asyn-131, asyn-134, asyn-142), by ELISA and surface plasmon resonance (SPR). For asyn-055 and asyn-058, counter-screen by ELISA was performed with beta- and gamma-synuclein.

The specificity of asyn-131mAb and asyn-134 mAb against truncated isoforms of aSyn has been analyzed by SPR using the mAbs captured by a Fc specific anti-rabbit polyclonal antibody on the sensor chip and biotinylated peptides corresponding to the C-termini of truncated aSyn (i.e. aSyn (aa 106-119) or aSyn (aa 106-122), respectively) as analytes. Biotinylated C-terminal elongated peptides (aSyn(aa 106-122) or aSyn(aa 106-124), respectively) have been used as negative control to demonstrate the specificity of the mAbs for the truncated C-terminal sequence. The asyn-142 mAb against aSyn phosphorylated at Serine-129 was analyzed similarly, whereas biotinylated peptides corresponding to Ser129-p phosphorylated or non-phosphorylated aSyn (i.e. aSyn(aa 122-135, pSer129) or aSyn(aa 122-135), respectively) were employed as analytes. Biotinylated peptides have been grafted with streptavidin to increase the sensitivity of the SPR analysis. Analyses have been performed on a Biacore B4000 instrument at 25°C according to a method described by Schräml and Biehl.[4]

Western blot validation of antibodies with recombinant aSyn

Protein expression and purification: Recombinant full-length aSyn was expressed from the pRT21 expression vector in BL21(DE3) competent Escherichia coli (E. coli) as previously described [5]. C-terminally truncated forms of aSyn(1-119), aSyn(1-121), and aSyn(1-122) were expressed in BL21-DE3-pLysS competent E. coli (plasmids courtesy of Prothena Biosciences, South San Francisco, CA, USA). Purification of aSyn strains was performed by periplasmic lysis, ion exchange chromatography, ammonium sulfate precipitation, and gel filtration chromatography as previously described [6, 7]. Polo like kinase 2 (PLK2) was expressed in BL21-DE3-pLysS competent E. coli, isolated via its His-tag and immediately used to phosphorylate purified aSyn. This was followed by standard ion exchange and gel filtration chromatography to separate phosphorylated from non-phosphorylated aSyn. The sequence of the expressed aSyn strains was verified by tryptic digestion followed by MALDI mass spectrometry (MS) or HPLC/ESI tandem MS for total mass was performed. Purity and monodispersity was determined by Coomassie blue or Silver staining of the SDS PAGE gel and analytical ultracentrifugation and the concentration was determined by the bicinchoninic acid (BCA) assay (Thermo Scientific) with bovine serum albumin as a standard. Dialyzed and lyophilized aSyn(1-121) was prepared by dialyzing the purified protein in a 2 kD Slide-A-Lyzer unit (Thermo Scientific, for max. 3 ml) against HPLC-water (VWR). 500 µg protein aliquots were pipetted into 1.5ml tubes, frozen on dry ice, and lyophilized for 2h using an Eppendorf concentrator (Eppendorf). Lyophilized samples were stored at -80°C until use.

SDS PAGE and WB analysis: Purified recombinant aSyn (~11 ng per lane) was loaded onto 4-12% SDS-PAGE Bis-Tris Gels (NuPAGE, Invitrogen Ref. NP0323BOX; MES running buffer, Invitrogen Ref. NP0002, non-reducing conditions) and transferred to nitrocellulose membranes (Midi Format 0.2um NC-Membran, BioRad Ref. 1704159) via Trans-Blot Turbo technology (BioRad, method “mixed MW”). Membranes were blocked for 15min in SuperBlock Solution (Thermo Scientific, Ref. 37515), followed by 15min in 5% non-fat-dry milk in 0.1% Tween/PBS at room temperature. Primary antibodies were diluted to 1ug/ml (exceptions: sc211 diluted to 0.1ug/ml, syn105 diluted to 0.01ug/ml, 4B12 diluted to 0.2ug/ml) in 0.5% non-fat-dry milk in 0.1% Tween/PBS solution and incubated overnight at 4°C. Membranes were washed with 0.1% Tween/PBS and subsequently incubated with the secondary antibodies goat anti-mouse IgG HRP (abcam Ref. ab6789) or goat anti-rabbit IgG HRP (abcam, ab6721), both diluted 1:25’000 in 0.5% non-fat-dry milk in 0.1% Tween/PBS solution for 1.5 hour at room temperature. Immunodetection was performed using the Lumi-

Light Western Blotting Substrate (Roche Ref. 12015200001) and Fusion FX7 Imaging System (Vilber).

Western blot validation of antibodies with human brain tissue extracts

Tissue processing: We adapted and largely modified with current technologies a previously published protocol by Iwai et al. [8] Frozen tissue blocks of midbrain containing the putamen from two postmortem human normal brains and two patients with DLB were manually sliced into sections of 40-70 mg in a cryostat (at max. -10°C) and tissue was immediately stored in Eppendorf tubes at -80°C until further processing. After quickly washing the tissue in PBS pH 7.2 (10x stock solution from BioRad 161-0780) containing 2x complete protease inhibitor cocktail (Roche, 11 873 580 001) and 1x phosphatase inhibitor cocktail set II (Calbiochem, 524625) for thawing and blood removal, it was immediately transferred to Precellys Lysing Kit CK14_0.5ml tubes (Ref: P000933-LYSK0-A) containing 1:4 (w/v) precooled 5 mM HEPES pH 8.0 (PanReac – AppliChem, Ref. A1069,0100) containing 320 mM Sucrose (Sigma-Aldrich 16104-1KG) and 2 mM Dithiothreitol (DTT, Invitrogen, Ref. NP0004) with 2x complete protease inhibitor cocktail and 1x phosphatase inhibitor cocktail set II (herein called HEPES/sucrose buffer). Subsequently, tissue was homogenized using a Precellys homogenizer (6500rpm, 4 x 20s, ~5-10°C in rotor compartment, art. no: 03119.200.RD000 ; Bertin Technologies SAS, France) with Cryolis cooling unit (filled with dry ice, art.no 05068.200.RD000; Bertin Technologies). Between the four homogenization steps, the tubes were incubated on ice for 5 minutes, while the tubes were centrifuged for 2 min at 4°C at 1000 x g after each homogenization. Tissue homogenates were transferred (without ceramic beads) to 1.5ml protein lobind tubes (Eppendorf 022431081), and centrifuged for 10min at 1000 x g at 4°C (Eppendorf Centrifuge, art. no. 5417R). The supernatant 1 (S1) was transferred in two equal aliquots in two centrifugal tubes (Beckman, art. no. 357448; 1.5ml-Polyallomer-Tubes) and centrifuged at 100’000 x g at 4°C for 60min (Beckman TL-100 ultracentrifuge with rotor TLA-55). This HEPES/sucrose-soluble fraction containing supernatant 2 (S2) was removed from the two pellet 2 (P2), pooled, and immediately frozen on dry-ice and stored at -80°C. P2 aliquots were washed by adding 125ul HEPES/sucrose buffer with 2x complete protease inhibitor cocktail and 1x phosphatase inhibitor cocktail set II, incubate for 10min on ice (w/o resuspending) and centrifuged at 100’000 x g at 4°C for 30min. The washed P2 samples were immediately frozen on dry-ice for at least 15 minutes or stored at -80°C.

SDS PAGE and WB analysis: Samples were thawed on ice, diluted 1:2 in 1x sample buffer (SB), containing 50ul 4x LDS sample buffer (Invitrogen, Ref NP0007), 20ul DTT, and 130ul DPBS 1x (Gibco 14190) and resuspended by pipetting ~25x up and down. The entire suspension was subsequently frozen at -80°C for at least 15min. Samples were thawed on ice and for loading 20 µl onto 4-12% SDS-PAGE Bis-Tris Gels (NuPAGE, Invitrogen Ref. NP0322BOX), the S2 samples were diluted with DPBS 1:4 and the P2 diluted 1:6, mixed with reducing SB as above and before loading to the gel cooked for 10min at 70°C and quickly spun down to remove drops from the lid. Electrophoresis was done with MES running buffer (Invitrogen Ref. NP0002). Content and quality of extracts were inspected for all samples by Coomassie blue and silver stainings of SDS PAGE electrophoresis gels. For WB, transfer was performed onto nitrocellulose membranes (0.45um, Thermo Scientific Ref. 88025) with the semi-dry method (BioRad, 1h, 20V; Novex Transferbuffer Ref. NP0006-1 with additional 20% methanol). For antigen retrieval, some membranes was cooked for 5min in 100ml 1x TBS (BioRad Ref. 170-6435) in a microwave oven at 400W. All membranes were blocked for 1h in SuperBlock Solution (Thermo Scientific, Ref. 37515), followed by 1h in 5% non-fat-dry milk in 0.1% Tween/PBS at room temperature. Primary antibodies were diluted to 1ug/ml (exceptions: sc211 diluted to 0.1ug/ml, syn105 diluted to 1, 0.1 and 0.01ug/ml, 4B12 diluted to 0.2ug/ml, 0018 diluted to 0.2ug/ml) in 0.5% non-fat-dry milk in 0.1% Tween/PBS solution and incubated under gentle shaking overnight at 4°C. Membranes were washed with 0.1% Tween/PBS and subsequently incubated with the secondary antibodies goat anti-mouse IgG HRP (abcam Ref. ab6789) or donkey anti-rabbit IgG HRP (Invitrogen, A16035), diluted 1:25'000 in case of goat anti-mouse and 1:10'000 in case of donkey anti-rabbit in 0.5% non-fat-dry milk in 0.1% Tween/PBS solution for 1.5 hour at room temperature. Immunodetection was performed using the Lumi-Light Western Blotting Substrate (Roche Ref. 12015200001) and Fusion FX7 Imaging System (Vilber).

References Online Resource methods:

1. Anderson JP*, et al.* Phosphorylation of Ser-129 is the dominant pathological modification of alpha-synuclein in familial and sporadic Lewy body disease. *J Biol Chem* **281**, 29739-29752 (2006).

2. Games D*, et al.* Axonopathy in an alpha-synuclein transgenic model of Lewy body disease is associated with extensive accumulation of C-terminal-truncated alpha-synuclein. *Am J Pathol* **182**, 940-953 (2013).

3. Games D*, et al.* Reducing C-terminal-truncated alpha-synuclein by immunotherapy attenuates neurodegeneration and propagation in Parkinson's disease-like models. *J Neurosci* **34**, 9441-9454 (2014).

4. Schraml M, Biehl M. Kinetic screening in the antibody development process. *Methods Mol Biol* **901**, 171-181 (2012).

5. Guerrero-Ferreira R*, et al.* Cryo-EM structure of alpha-synuclein fibrils. *Elife* **7**, (2018).

6. Huang C, Ren G, Zhou H, Wang CC. A new method for purification of recombinant human alpha-synuclein in Escherichia coli. *Protein Expr Purif* **42**, 173-177 (2005).

7. Luk KC*, et al.* Exogenous alpha-synuclein fibrils seed the formation of Lewy body-like intracellular inclusions in cultured cells. *Proc Natl Acad Sci U S A* **106**, 20051-20056 (2009).

8. Iwai A*, et al.* The precursor protein of non-A beta component of Alzheimer's disease amyloid is a presynaptic protein of the central nervous system. *Neuron* **14**, 467-475 (1995).

Online Resource Table 1: Different donors included in this study. Abbreviations: CD: clinical diagnosis; CERAD: CERAD age-related score for neuritic plaques; Exp: Experiments: 1) FFPE sections used for multiple labeling experiments; 2) Snap-frozen sections used for CARS microscopy; 3) WB analyses. Three of these donors (indicated by *) were also studied in the study by Shahmoradian et al. (Ref 67). The identifier of these donors in this study is added to the table. PDD: PD with dementia; AD: Alzheimer's Disease

**ID**	**CD**	**Age at death**	**Sex**	**Braak LB score**	**Braak NFT score**	**CERAD score**	**Age of diagn.**	**Age at onset dementia**	**Exp**	**Figure**	**Donor ID in REF67**
PD1	PDD	80	M	6	1	B	55	73	1	3A,E, S5, 7	
PD2*	PDD	72	M	6	1	O	64	69	1	1A	**J**
PD3	PD	72	M	6	1	A	57	-	1	-	
PD4*	PDD	80	M	6	1	O	69	77	1	1B; 6B,D; S3A,C, S5, S7A, 7	**L**
PD5*	PD	77	M	6	2	O	59	-	1	3B,C	**A**
PD6	PD+ AD	74	M	6	4	B	64	73	1	2B; 4A,B,D; 6A,C; S5, S6	
PD7	PDD	66	F	6	1	A	57	64	1	2A; 3D; 4C,E;6E; S3D; S2;S4	
PD8	PDD	87	M	6	3	C	77	86	1	2C	
PD9	PD	67	F	6	1	B	60	-	2	-	
PD10	PDD	81	M	6	2	O	72	79	2	5C	
PD11	PDD	74	M	5	3	O	67	71	2	5D	
PD12	PDD	76	M	6	1	B	66	71	2	5A, B	
PD13	PDD	83	M	6	1	B	78	80	1,2	S5	
PD14	PD	90	F	3	2	A	85	-	1	S8, 7	
PD15	PDD	85	F	4	2	C	74	82	1	-	
PD16	PDD	81	M	5	1	O	64	78	1	S8	
PD17	PDD	73	M	5	1	O	69	72	1	S8	
DLB1	DLB	90	M	6	1	B	79	79	3	S1	
DLB2	DLB	71	M	6	3	B	63	63	3	S1	
iLBD 1	C	93	F	3	2	O	-	-	1	S8, 7	
iLBD 2	C	86	F	3	3	A	-	-	1	S8, 7	
iLBD 3	C	98	M	3	2	O	-	-	1	S8, 7	
iLBD 4	C	79	M	3	2	B	-	-	1	7	
iLBD 5	C	70	M	3	0	O	-	-	1	7	
iLBD 6	C	82	F	3	1	O	-	-	1	7	
iLBD 7	C	75	M	5	1	C	-	-	1	S8	
C1	C	83	F	0	3	A	-	-	1	S6A	
C2	C	80	M	0	1	B	-	-	1	-	

C3	C	89	F	0	1	A	-	-	1	S6B, 7	
C4	C	84	F	0	1	O	-	-	1	-	
C5	C	81	M	0	2	O	-	-	1	-	
C6	C	70	F	0	2	A	-	-	1	-	
C7	C	76	F	0	2	O	-	-	1	7	
C8	C	82	F	0	2	O	-	-	1	7	
C9	C	78	F	0	3	A	-	-	3	S1	
C10	C	85	F	0	1	A	-	-	3	S1	

Online Resource Table 2: Summary of antibodies used in multiple labeling experiments in this study using direct and indirect detection methods. Exp: experiment. 1) light microscopy; 2) multiple labeling of aSyn epitopes; 3) multiple labeling of aSyn epitopes with 119CTT; 4) multiple labeling of aSyn epitopes, validation set; 5) multiple labeling PTMs and cytoskeletal markers; 6) multiple labeling PTMs + ER; 7) multiple labeling PTMs and mitochondria. *: biotinylated antibody used in multiple labeling protocols. When PTM-specific is indicated as targeted epitope, the antibody was raised to specifically recognize PTM protein variants of aSyn (see text Online Resource methods). Details on the initial characterization of these antibodies are presented in the Online Resource Methods, Online Resource Fig. 1 and Online Resource Table 6 and. **Abbreviations:** AB ID: antibody identifier; Exp: experiment; Ref: reference for additional information on AB characterization; d.l.: antibody directly labeled with fluophore

Targeted epitope	AB ID (Cat #)	Source	Host	Exp	Fluorochromes	STED wavelength	Ref	Figure
**aSyn antibodies**								
Ser129-p aSyn (phospho-Ser129 in aSyn; PTM-specific)	11A5	Prothena	M	1,2,5,6,7	Alexa 488(d.l.)/Alexa 488	592	[3]	1, 2, S1, S2, S3, S4, S7A
Ser129-p aSyn (phospho-Ser129 in aSyn; PTM-specific)	asyn-142 (same as 7E2)	Roche	R	1,4,5,6,7	Alexa 488 (d.l.)/Alexa 488	592	[1]	4, 5, 6, S2, S4, S6, S7B. S8
122CTT aSyn (aSyn truncated at aa122; PTM-specific)	syn105	Prothena	R	1,2*,5*, 6*,7	Alexa 547<br>Alexa 594/Abberior 635p	660<br>775	[23]	1, 2, S1, S3, S7A
122CTT aSyn (aSyn truncated at aa122; PTM-specific)	asyn-134	Roche	R	1,4*,5*, 6*,7	Alexa 547<br>Alexa 594/Abberior 635p	660<br>775		5, S2, S7B, S8
119CTT aSyn (aSyn truncated at aa119; PTM-specific)	asyn-131	Roche	R	1,3,5,7	Alexa 647 (d.l.)	775		1, 2, S1, S3, S8
aSyn NT (40-55)	23E8	Prothena	M	1,4	Alexa 647 (d.l.)	775		1, 2, S1, S3, S7
aSyn NAC domain (presumed conformational epitope within aa61-95)	asyn-055	Roche	R	1,2	Alexa 488<br>Li-Cor 680	592<br>775		1, 2, S1, S7
aSyn NAC domain (presumed conformational epitope within aa61-95)	asyn-058	Roche	R	1,4	Li-Cor 680	775		S2
aSyn CT (118-126)	5C1	Prothena	M	1,2	Alexa 594	775	[24]	1, 2. S1. S3. S7
aSyn CT (121-125)	SC-211	Santa Cruz	M	1,4	Alexa 594	775	[26]	S2
KM-51 (recombinant full-length aSyn; unknown epitope within aa1-140)	MONX1 0738	Monosan	M	1	- (only light microscopy)	-		S1

Online Resource Table 3: WB detection of recombinant aSyn protein variants by different antibodies (Summary of Online Resource Fig. 1B). X: detection; - : no detection

		Antibody									
		11A5	asyn-142	Syn105	asyn-134	asyn-131	23E8	asyn-055	asyn-058	5C1	SC211
aSyn Variant	aSyn 1-140	-	-	-	-	-	X	X	X	X	X
	aSyn 1-119	-	-	-	-	X	X	X	X	-	-
	aSyn 1-122	-	-	X	X	-	X	X	X	-	-
	Ser129-p aSyn	X	X	-	-	-	X	X	X	X	X

Online Resource Table 4: Number of structures scanned per patient and locations for peak intensities for different aSyn epitopes in the semi-quantitative measurement of line profiles. Values represent the distance of peak intensities relative to the origin of the LB (with values of 0 representing the LB origin, 50 the peripheral border, Fig. 2F)

LB #	Donor	DAPI	NAC	NT	CTT	Ser129-p	CT
1	PD1	9	15	15	28	35	45
2	PD1	19	7	3	22	39	48
3	PD1	4	34	35	25	38	46
4	PD2	4	23	24	37	40	47
5	PD2	9	24	19	20	35	45
6	PD4	3	10	10	33	40	43
7	PD4	2	2	34	1	32	46
8	PD4	0	14	27	24	33	40
9	PD4	20	3	3	25	40	46
10	PD5	3	0	1	1	30	37
11	PD5	4	47	48	24	39	47
12	PD5	16	14	11	39	37	42
13	PD5	17	2	2	46	47	36
14	PD5	25	1	4	30	35	41
15	PD5	6	17	17	31	39	41
16	PD5	6	16	3	34	38	46
17	PD6	1	19	20	21	36	45
18	PD6	2	16	12	29	35	42
19	PD6	18	16	10	1	37	43
20	PD6	11	46	48	34	39	48
21	PD6	12	47	48	37	41	48
22	PD6	0	8	0	34	37	46
23	PD7	4	13	46	21	38	41
24	PD7	0	1	1	36	44	47
25	PD13	5	43	43	32	38	46
26	PD13	3	17	22	17	27	46
27	PD13	20	5	11	16	39	44
28	PD13	10	9	11	15	35	38
29	PD13	1	2	5	26	38	47
30	PD13	18	1	7	30	37	42

Online Resource Table 5: Different localization of peak intensities between aSyn epitopes. Overview of Dunn's corrected p-values of multiple comparisons. Ns = not significant; *: p<0.05; **p<0.01; ***p<0.001

	NAC	NT	122CTT	Ser129-p	CT
**NAC**	**X**	1,00	<0.001***	<0.001***	<0.001***
**NT**	Ns	**X**	Ns	0.008**	<0.001***
**122CTT**	<0.001***	Ns	**X**	0.025*	<0.001***
**Ser129-p**	<0.001***	0.008**	0.025*	**X**	0.05*
**CT**	<0.001***	<0.001***	<0.001***	0.05*	**X**

Online Resource Table 6. Specifications of novel aSyn mAbs generated at Roche. Specific binding of aSyn Mabs was assessed by ELISA, custom-made PepStarTM peptide microarrays (JPT, Berlin, Germany), or SPR. For ELISA, the full length human recombinant αSyn, βSyn or γSyn proteins, indicated fragments or peptides were coated or linked by biotin tag on an ELISA plate, and incubated with primary antibody. Primary antibody was detected by anti-rabbit secondary antibody with a colorimetric readout (OD, optical density). For the peptide array, peptide sequences had a length of 15 amino acid residues and were designed to cover the whole sequence of human aSyn. Neighboring peptides had an overlapping sequence of 11 amino acids. Primary antibodies were hybridized with the array and detected by fluorescently labelled anti-rabbit IgG antibodies following the manufacturers' instruction. For SPR data see further Online Resource Fig. 1A. Binding rate indicates the binding strength of the peptide towards the mAb at the end of the association phase; rec., recombinant; RU, relative unit

Antibody specifications (part 1)

**Epitope**	**mAb**	**OD on ELISA / SPR binding late (RU) in kinetic screening mode**
aSyn 119CTT	asyn-131 (rabbit)	aSyn(106-119): 1.119 / 324<br>aSyn(106-122): 0.088 / 14
aSyn 122CTT	asyn-134 (rabbit)	aSyn(111-122): 1.368 / 523<br>aSyn(111-124): 0.075 / 1
aSyn pSer129	asyn-142 (rabbit)	aSyn(122-135, pSer129): 1.209 / 152<br>aSyn(122-135): 0.145 / 3

Antibody specifications (part 2)

Epitope	mAb	hu rec. aSyn Monomer Binding EC50 (ng/ml)<br><br>hu rec. aSyn, aa1-60 (OD)<br><br>hu rec. aSyn, aa96-140 (OD)	Protein array for full length α-, β-, or γSyn<br><br>Peptide array-based epitope mapping, immunoreactivity for indicated peptides<br><br>Overall conclusion
NAC	asyn-055 (rabbit)	1508.0<br><br>0.0<br><br>0.0	Binds to αSyn but not βSyn or γSyn<br><br>aa65-79 (weak), aa93-107, aa97-111 (weak)<br><br>Based on ELISA and epitope mapping, presumable conformational epitope in NAC region
NAC	asyn-058 (rabbit)	1167.7<br><br>0.0<br><br>0.0	No binding to αSyn, βSyn, γSyn<br><br>No detection of imunoreactivity to any peptide<br><br>Based on clear result in ELISA, presumable conformational epitope in NAC region

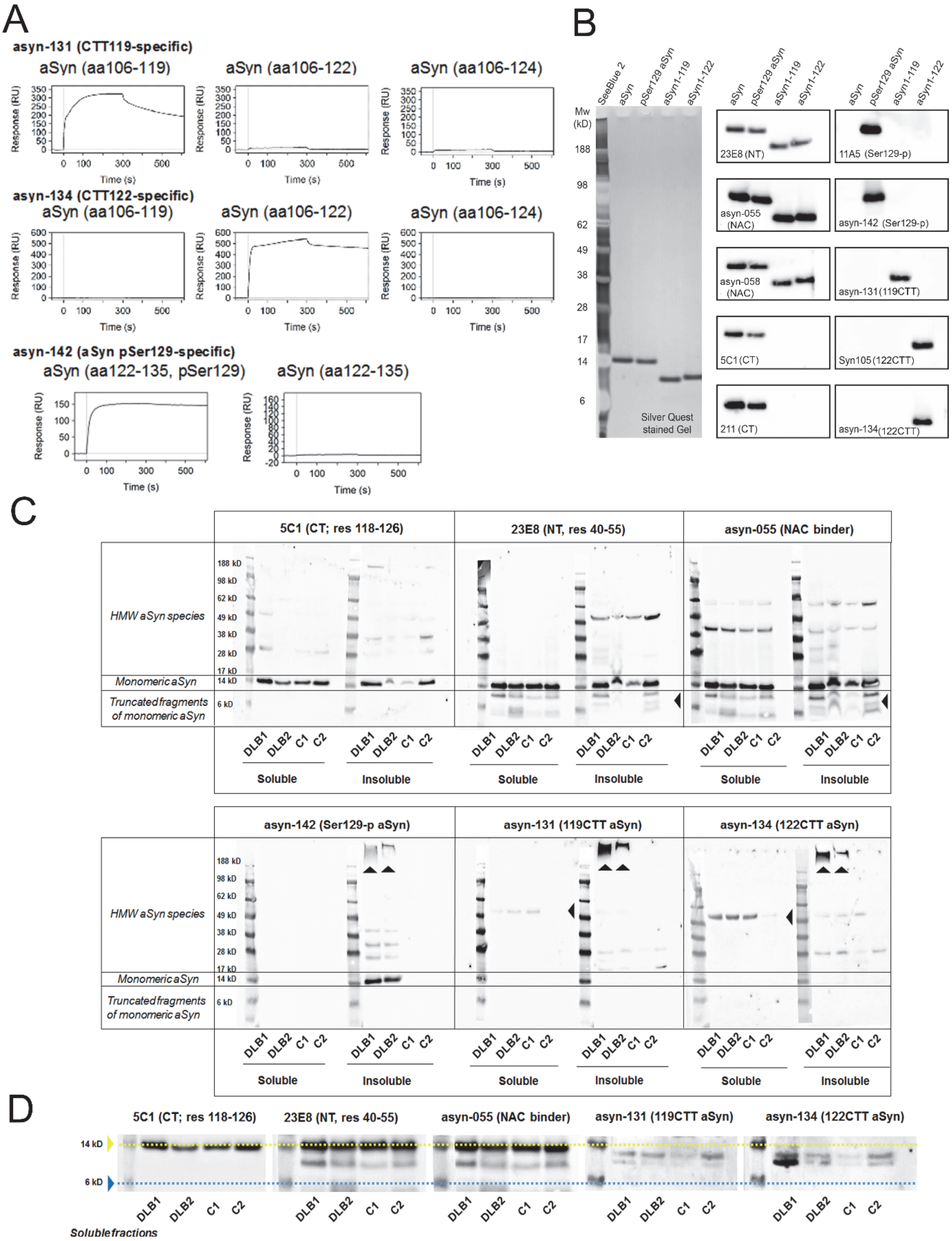

A
asyn-131 (CTT119-specific)
aSyn (aa106-119)
aSyn (aa106-122)
aSyn (aa106-124)
asyn-134 (CTT122-specific)
asyn-142 (aSyn pSer129-specific)
aSyn (aa122-135, pSer129)
aSyn (aa122-135)
Response (RU)
Time (s)
B
SeeBlue 2
aSyn
pSer129 aSyn
aSyn1-119
aSyn1-122
Mw (kD)
Silver Quest stained Gel
23E8 (NT)
11A5 (Ser129-p)
asyn-055 (NAC)
asyn-142 (Ser129-p)
asyn-058 (NAC)
asyn-131(119CTT)
5C1(CT)
Syn105(122CTT)
211 (CT)
asyn-134(122CTT)
C
5C1 (CT; res 118-126)
23E8 (NT, res 40-55)
asyn-055 (NAC binder)
asyn-142 (Ser129-p aSyn)
asyn-131 (119CTT aSyn)
asyn-134 (122CTT aSyn)
HMW aSyn species
Monomeric aSyn
Truncated fragments of monomeric aSyn
DLB1
DLB2
C1
C2
Soluble
Insoluble
D
Soluble fractions

Online Resource Fig. 1: Characterization of antibodies against Ser129-p aSyn, CTT aSyn, and aSyn domains, and their biochemical detection of aSyn species. a: SPR results, demonstrating specificity of asyn-131 and asyn-134 for peptides corresponding to the CT of 119CTT (aa 106-119) and 122CTT aSyn (aa 106-122), respectively. The asyn-142 mAb against Ser129-p aSyn was analyzed similarly, using peptides corresponding to Ser129-p (aa 122-135, pSer129) versus non-phosphorylated (aa 122-135) aSyn. RU: relative units **b:** WB analysis on recombinant proteins. As expected, 23E8 (aa40-55), asyn-055, and asyn-058 (both with presumed epitopes within the NAC region) bind all analyzed forms of aSyn. Binding of CT antibodies 5C1 (epitope in aa118-126) and 211 (epitope within aa121-125) was abolished for aSyn1-119 and aSyn1-122 whereas full-length aSyn and Ser129-p aSyn were recognized as expected. Note the strong specificity of 11A5 and asyn-142 for pSer129 aSyn, of asyn-131 to aSyn1-119, and of syn105 and asyn-134 to aSyn1-122 with lack of binding to other forms of aSyn. **c:** WB results on HEPES-soluble and insoluble putamen fractions from 2 controls and 2 DLB patients. 14kDa monomeric full-length aSyn is detected by antibodies against different aSyn domains. Antibodies against Ser129-p and CTT aSyn show detection of insoluble, very HMW aSyn species in DLB patients but not in controls (arrowheads). **d:** Truncated aSyn fragments (<14kDa) but not full-length monomeric aSyn are detected by antibodies against 122CTT and 119CTT in soluble fractions of DLB patients and controls after heat-induced epitope retrieval. More details on the characterization of the novel antibodies are provided in the Online Resource Methods paragraphs.

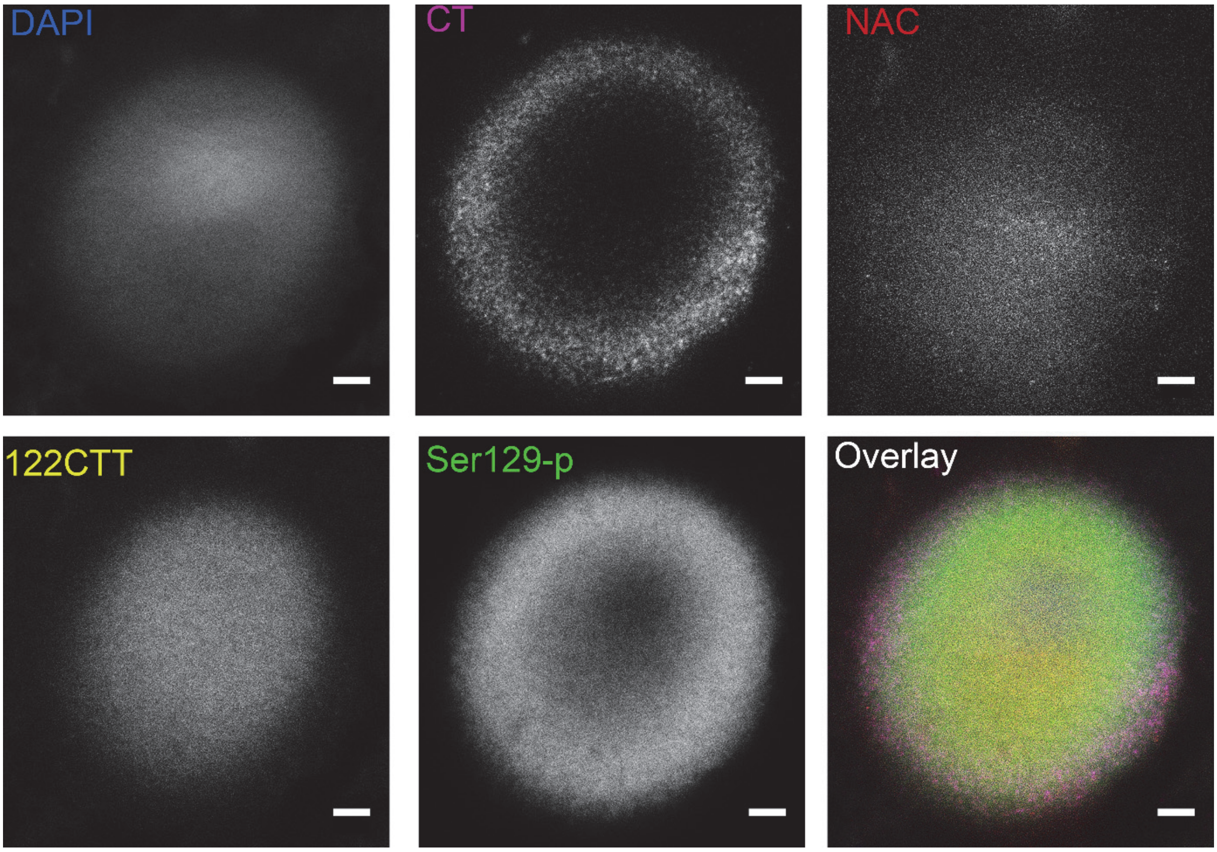

Online Resource Fig. 2: Onion skin-type organization of LBs as observed with different antibodies (validation set). Antibodies used: CT: 211; NAC: asyn-58; 122CTT: asyn-134; Ser129-p: asyn-142. Scale bar: 2µm

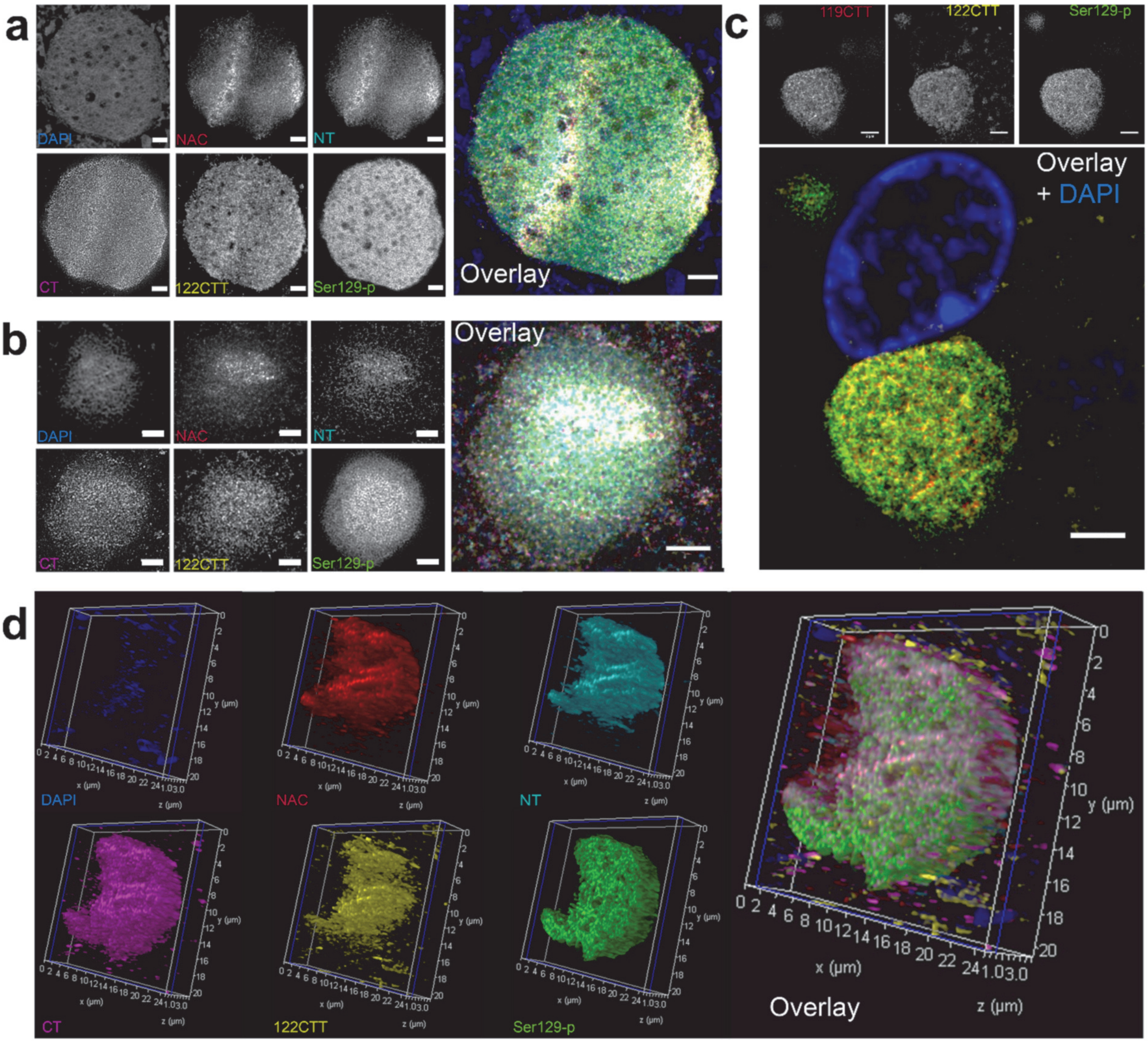

Online Resource Fig. 3: Distribution patterns of aSyn PTMs and domains in nigral expansive-appearing aSyn inclusions and cortical LBs. Antibodies shown: CT: 5C1; NT: 23E8; NAC: asyn-55; 122CTT: syn105; Ser129-p: 11A5. **a, b, c:** Representative deconvoluted STED images of nigral inclusions without ring-shape in the SN (**a**), transentorhinal cortex (**b**). and hippocampus (**c**) of patient PD4. The distribution of immunoreactivities was generally more diffuse, although CT and Ser129-p aSyn immunoreactivity at the edges of the structure generally sharply outlined the inclusion. **d.** 3D reconstruction of an uniform expansive-appearing inclusion body in the SN based on deconvolved CSLM images, taken in patient PD7. **A-C:** Scale bar = 2 μm

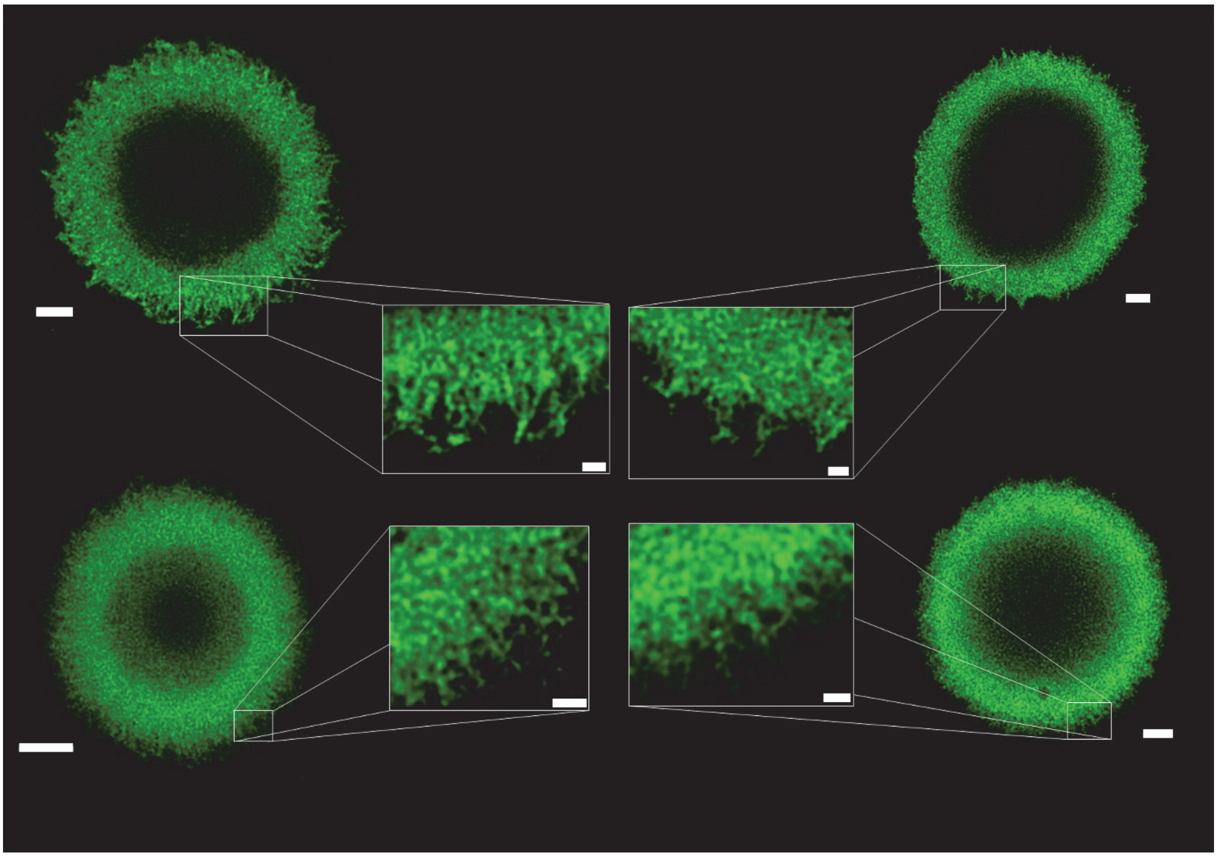

Online Resource Fig. 4: Gallery of radiating Ser129-p immunoreactivity patterns at the periphery of nigral LBs. STED deconvolved images taken in the SN of different PD patients using 11A5 and 142. Scale bars main Figure = 2μm; scale bars insets = 0.5μm

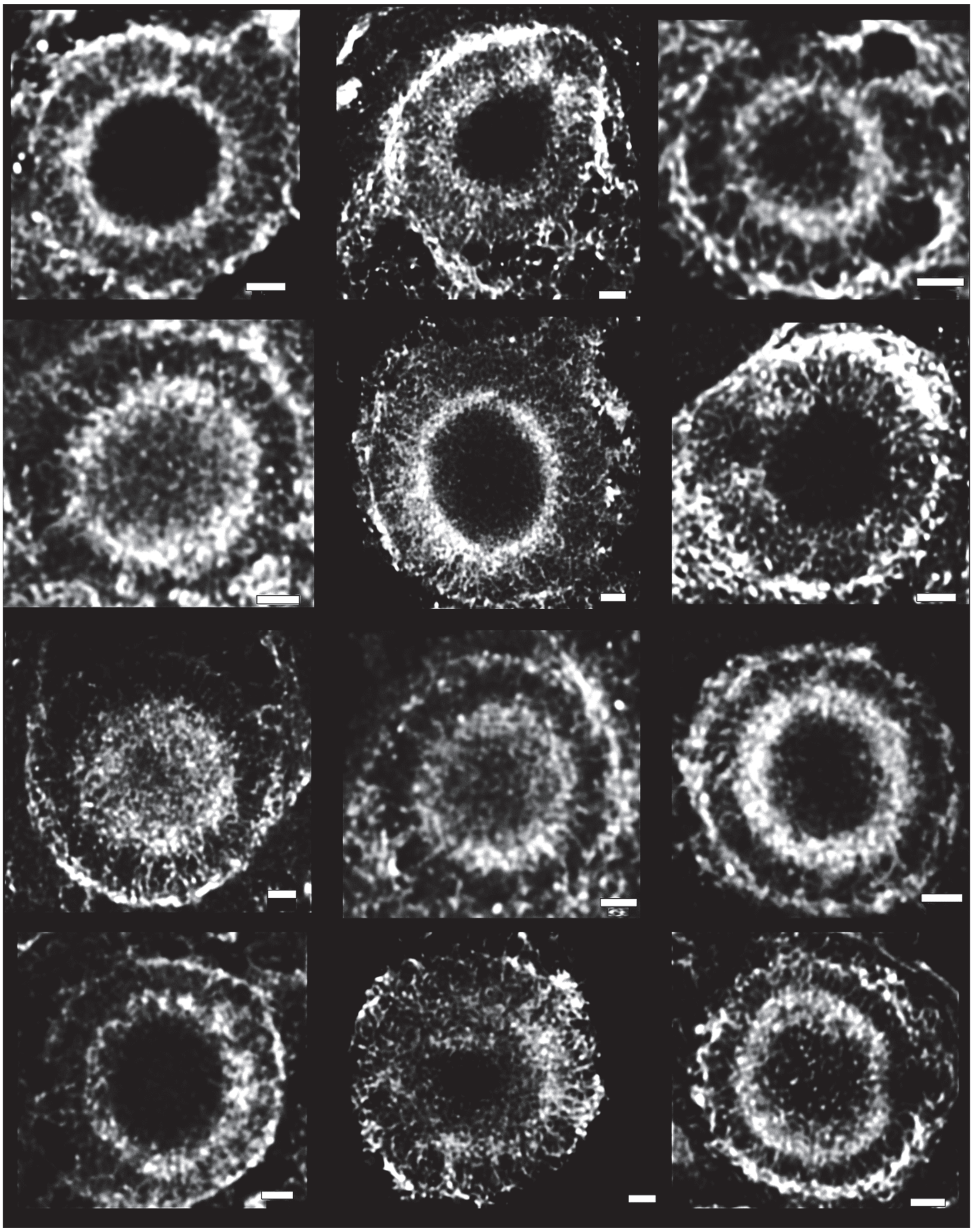

Online Resource Fig. 5: Gallery of 'wheel-like' neurofilament structures in nigral LBs immunopositive for Ser129-p aSyn (not shown). Deconvolved CSLM images captured in the SN of various patients (indicated in Online Resource Table 2). Scale bars = 2μm.

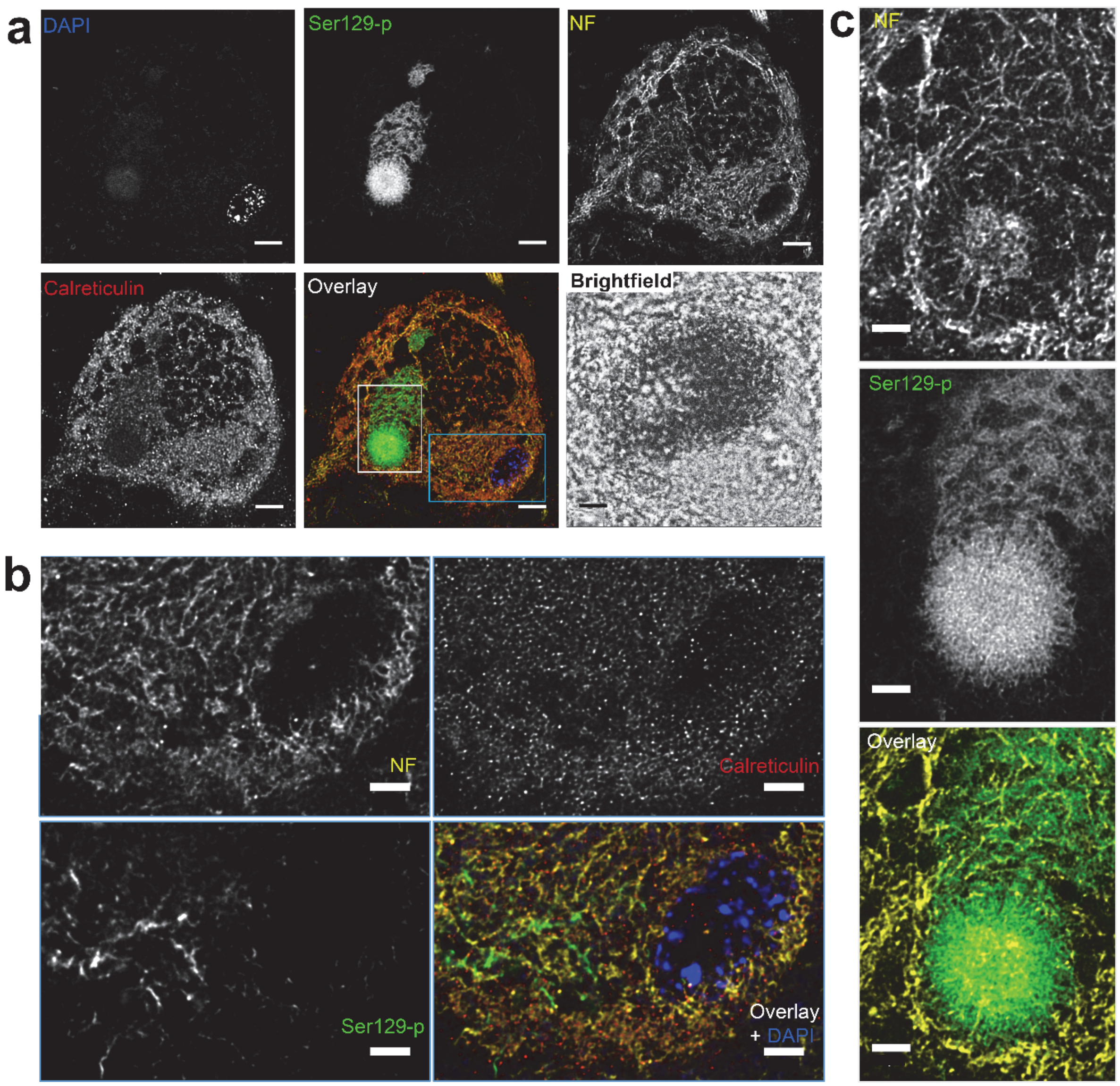

Online Resource Fig. 6: Cytoplasmic Ser129-p aSyn+ network profiles shows only partial co-localization with other intracytoplasmic networks. aSyn antibody shown: asyn-142. Images were taken in a dopaminergic neuron the SN of a PD patient containing a combination of uniform inclusion and a small LB with ring-structure. **a:** Overview: deconvolved CSLM images of the entire neuronal soma showing different intracytoplasmic networks reactive for calreticulin (ER marker), neurofilament and Ser129-p aSyn. Note the limited availability of epitope in the area containing neuromelanin (visible in brightfield image as dark material). **b:** Detailed view on the area in the blue square indicated in **a**. Deconvolved STED images showing limited co-localization between markers for intracytoplasmic networks of neurofilament. **c:**

Detailed view of the inclusion (area indicated in the grey square in **a**). The spherical ring-shaped LBs shows 1) radiating immunoreactivity patterns for Ser129-p aSyn; 2) a cage-like framework of neurofilament. NF: intermediate neurofilament. **a:** Scale bar = 5µm; **b, c**: scale bar = 2 µm

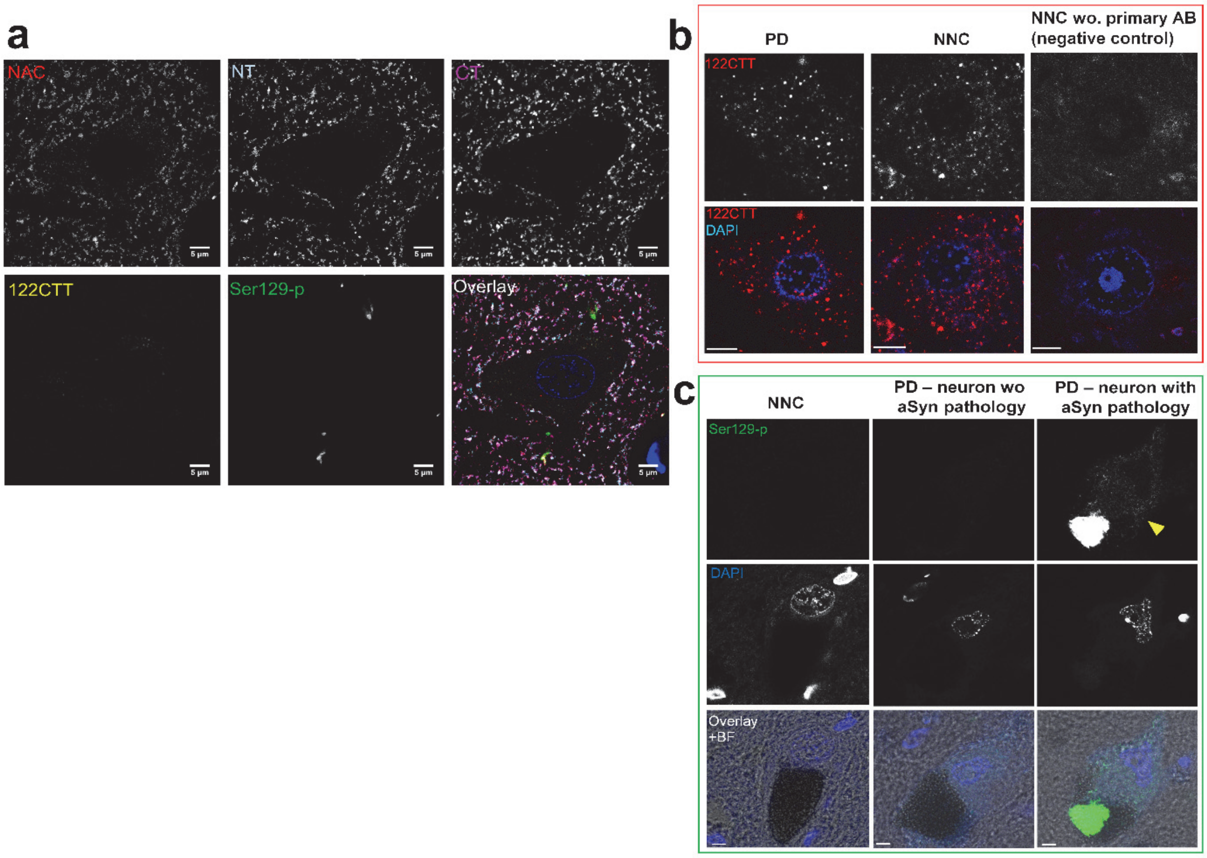

Online Resource Fig. 7: Subcellular reactivity patterns for antibodies against different aSyn domains, CTT and Ser129-p aSyn in brains without Lewy pathology. Antibodies shown: CT: 5C1; NAC: asyn-55; NT: 23E8; 122CTT: syn105 (**a**) and syn-134 (**b**), Ser129-p: 11A5 (**a**) and asyn-142 (**b**). **a:** Prominent neuropil staining patterns in the CA2 of the hippocampus consistent with synaptic-like profiles in a PD patient is observed for antibodies against different aSyn domains but absent in antibodies against proteoforms CTT and Ser129-p aSyn. **b:** In experiments with higher antibody concentrations, dot-like pattern of 122CTT aSyn was visible in cell bodies both in PD patients and brains without Lewy pathology (NNC = non-neurological control). This pattern was not observed in negative controls lacking the

primary antibody. Images taken in the hippocampus. **c:** a perinuclear Ser129-p positive network (indicated by yellow arrowhead) was only observed in certain cells in PD patients – often associated with the presence of an inclusion - and not in non-demented controls. Raw CSLM images that were scanned, processed and visualized in the same way. Images taken in neuromelanin-containing cells in the SN. **a,b:** Scale bar = 5µm. Abbreviations: NNC = non-neurological control; BF: brightfield

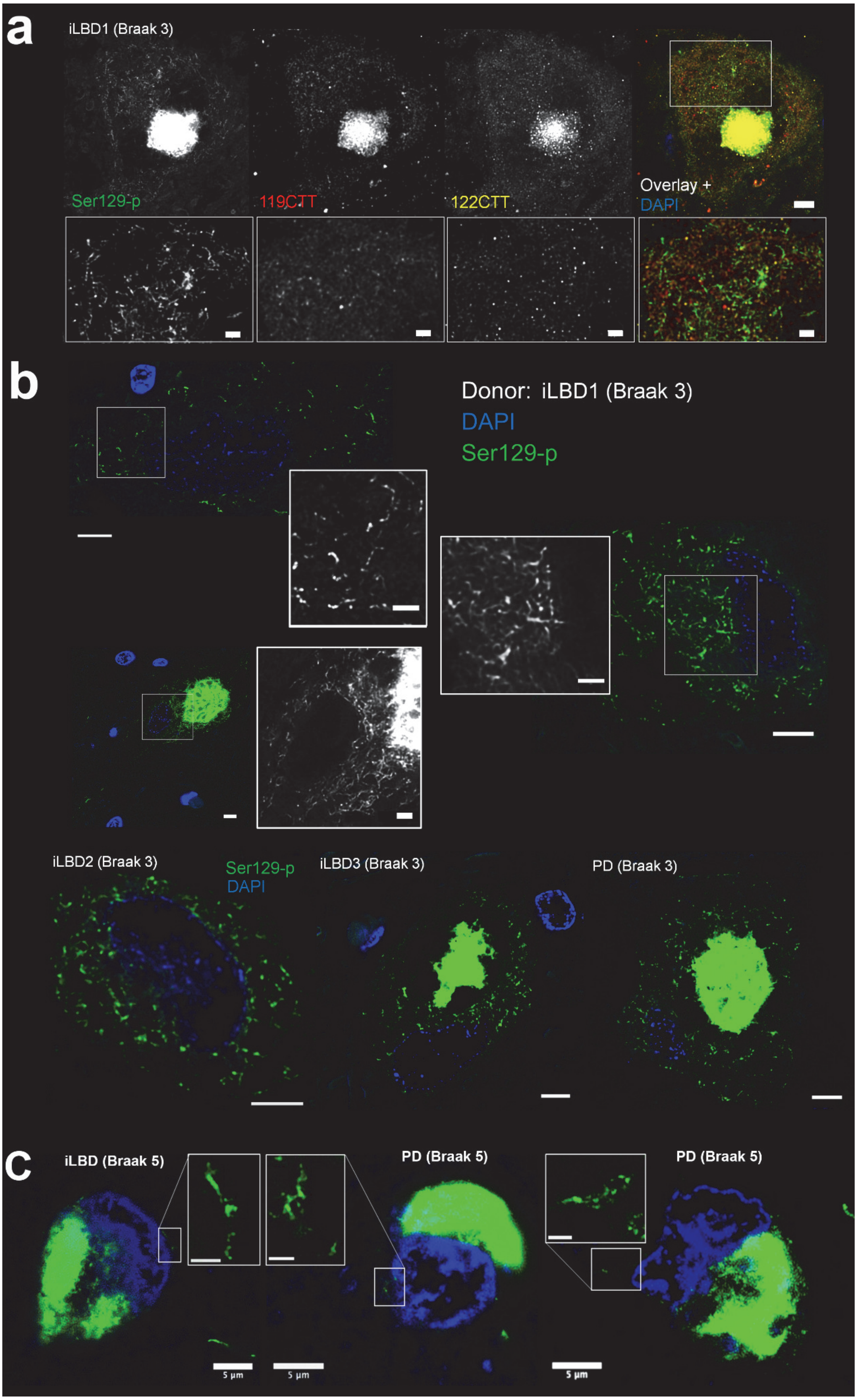
a
iLBD1 (Braak 3)
Ser129-p
119CTT
122CTT
Overlay +
DAPI
b
Donor: iLBD1 (Braak 3)
DAPI
Ser129-p
iLBD2 (Braak 3)
Ser129-p
DAPI
iLBD3 (Braak 3)
PD (Braak 3)
c
iLBD (Braak 5)
PD (Braak 5)
PD (Braak 5)
5 µm
5 µm
5 µm

Online Resource Fig. 8: cytoplasmic Ser129-p aSyn+ profiles in donors with iLBD and PD patients. Antibodies shown: 119CTT: asyn-131; 122CTT: asyn-134; Ser129-p: asyn-142. **a:** Ser129-p aSyn+ network is observed in the SN of donors with iLBD, but is not labeled by antibodies against 119CTT or 122CTT aSyn. **b:** More detailed view on some of the Ser129-p aSyn immunopositive perinuclear networks in neuromelanin-containing dopaminergic neurons with and without apparent inclusions in different Braak 3 iLBD and PD donors. Note that some of these features were very subtle in cells without inclusion (top left) as opposed to the dense network that is visualized in neurons with expansive-appearing inclusion bodies. **c:** Examples of Ser129-p aSyn immunoreactive networks in the temporal middle gyrus of Braak 5 iLBD and PD patients. Deconvolved CSLM images. **a:** Upper row: scale bar = 5 µm; lower row: scale bar = 2 µm; **b:** main image: scale bar = 5 µm; inset: scale bar = 2 µm; **c:** scale bar = 5 µm

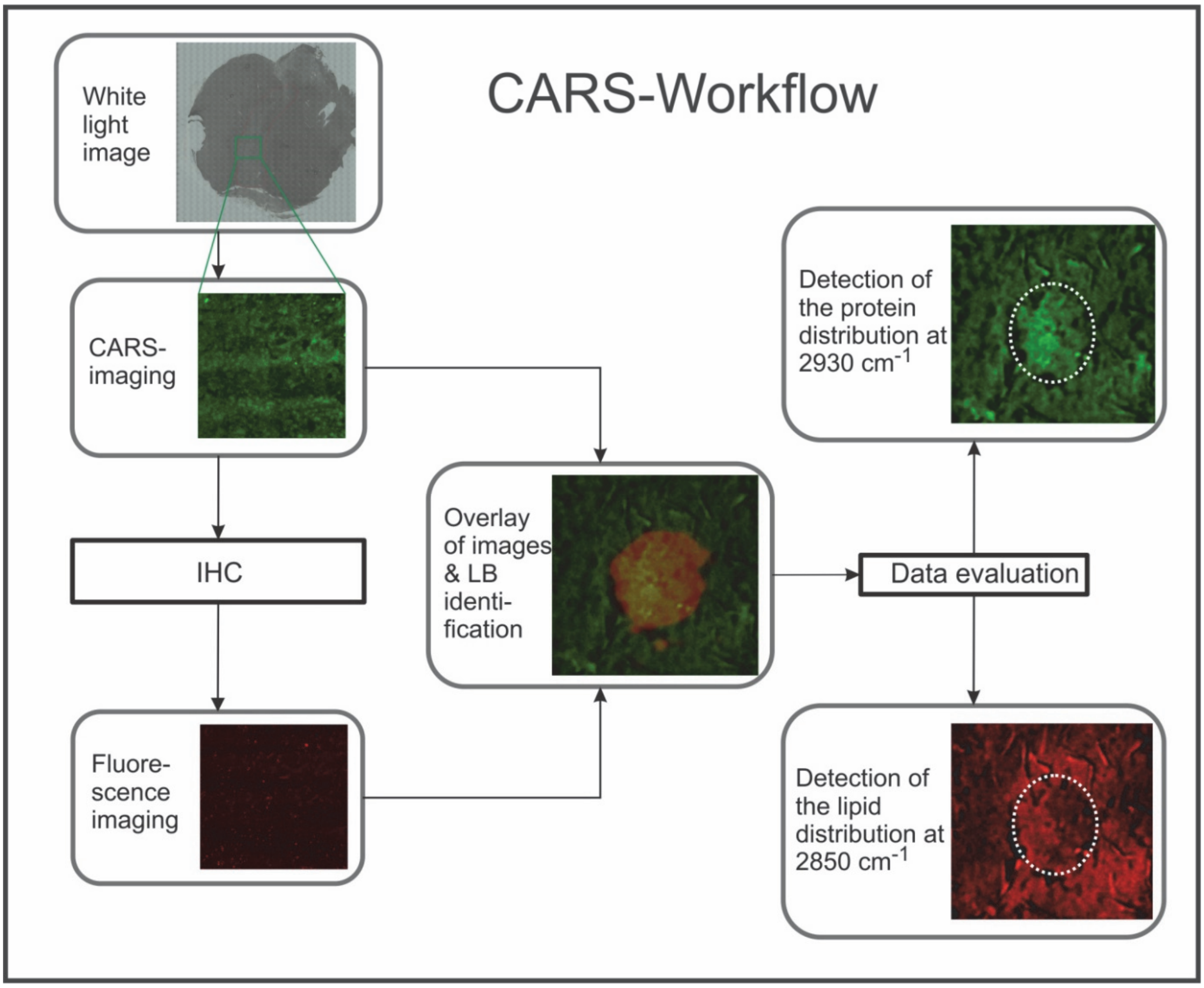

Online Resource Fig. 9: CARS-Workflow for the detection of the lipid and protein distribution in Lewy bodies. White light images were measured to detect the SN. The CARS-intensity was measured as overview images. On the same slide an immunohistological staining was performed and the fluorescence was detected for aSyn and Ser129-p aSyn. The images were manually overlaid. LBs were identified and small regions-of-interest (ROIs) were extracted. These ROIs were evaluated and the protein and lipid distribution of aSyn-positive inclusions was detected

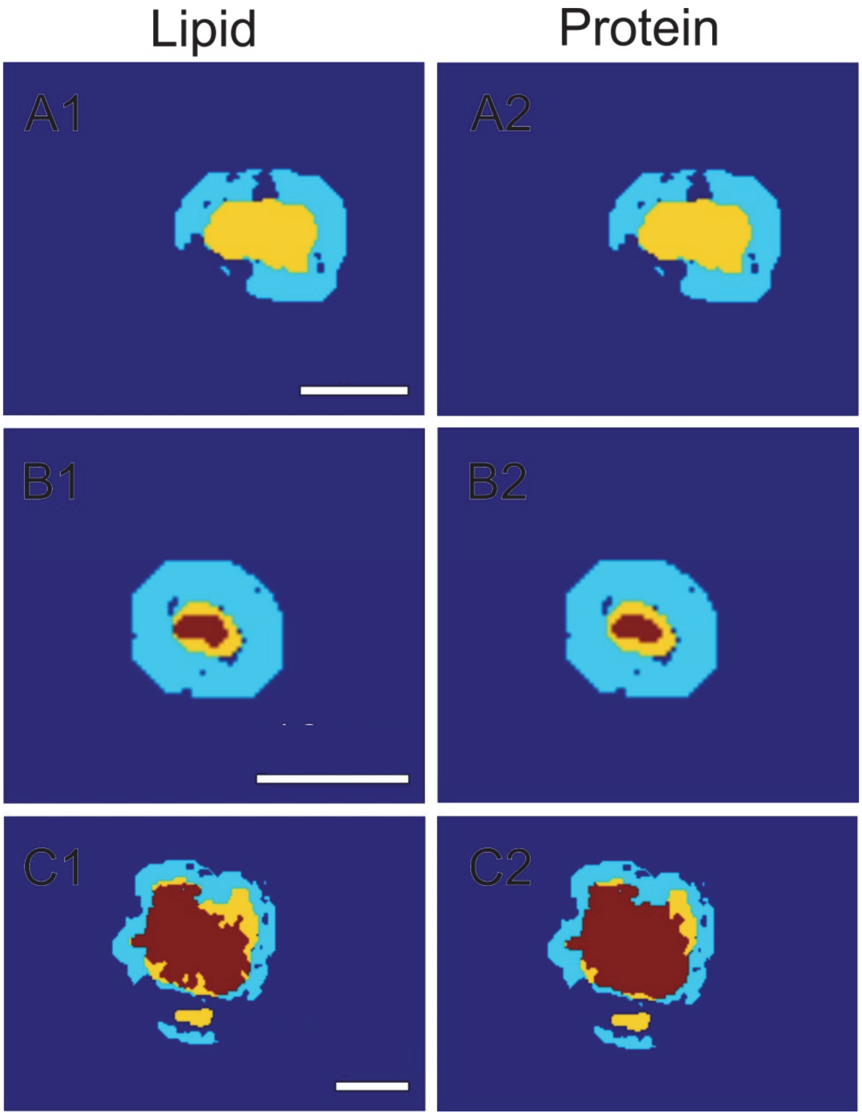

Online Resource Fig. 10: Evaluation of the centralization of lipids and proteins by CARS. The LBs were identified by aSyn staining (yellow) in the CARS-intensity images. For an objective evaluation of CARS intensity of the LBs, the mean CARS intensity of the direct surrounding (A, cyan), a donut with a width of 3.5 µm, was compared with the CARS-intensity of each LB pixel (A, yellow). CARS-pixel-intensities higher than 1.4 times the mean CARS-intensity of the surrounding were defined as higher protein/lipid content. The ratio between the CARS-pixel-intensities of the LB and the mean CARS intensity of the surrounding were calculated and areas with higher protein/lipid content were marked in red. Areas without CARS intensity (holes) were excluded by intensity thresholding in the first step. Scale bars = 10 µm

Legends for Online Resource video files:

Online Resource Video 1: 3D reconstruction based on deconvolved CSLM images revealing the distribution of Ser129-p, 119CTT and 122CTT aSyn in an onion skin-type LB in the SN of patient PD7

Online Resource Video 2: 3D reconstruction based on deconvolved CSLM images revealing the distribution of Ser129-p, 119CTT and 122CTT aSyn in another onion skin-type LB in the SN of patient PD6

Online Resource Video 3: 3D reconstruction based on deconvolved CSLM images revealing the distribution of antibodies directed against NT, NAC domain and CT in onion skin-type LBs in the SN of patient PD8

Online Resource Video 4: 3D reconstruction of an entire onion skin-type LB based on deconvolved CSLM images, showing distribution patterns of antibodies against different PTMs and domains of aSyn (captured in the SN of patient PD1).

Online Resource Video 5: 3D reconstruction based on deconvolved CSLM images of an aSyn inclusion without ring-shape, showing the distribution patterns of antibodies against different PTMs and domains of aSyn in the SN of patient PD8

Online Resource Video 6: 3D reconstruction based on deconvolved STED images showing the cage-like framework formed by Ser129-p aSyn and cytoskeletal components at the periphery of an onion skin-type LB in the SN of patient PD7

Online Resource Video 7: z-stack visualization of a cytoskeletal framework at the periphery of nigral onion skin-type LBs in the SN of patient PD7 (deconvolved CSLM images)

Online Resource Video 8: z-stack visualization of a wheel-like structure of neurofilaments at the periphery of nigral onion skin-type LBs in the SN of patient PD6 (deconvolved CSLM images)

Online Resource Video 9: 3D reconstruction based on deconvolved CSLM images showing cytoplasmic Ser129-p aSyn+ network profiles in an iLBD donor with Braak Stage 3 (iLBD1)

Online Resource Video 10: 3D reconstruction based on deconvolved STED images showing localization of a CTT-reactive punctae at the outer membrane of a mitochondrion immunopositive for VDAC/Porin (captured in the hippocampus of patient PD4).
